# A New Blood-Based Epigenetic Diagnostic Biomarker Test (EpiSwitch^®®^ NST) with High Sensitivity and Positive Predictive Value for Colorectal Cancer and Precancerous Polyps

**DOI:** 10.3390/cancers17030521

**Published:** 2025-02-04

**Authors:** Ewan Hunter, Heba Alshaker, Cicely Weston, Mutaz Issa, Shekinah Bautista, Abel Gebregzabhar, Anya Virdi, Ann Dring, Ryan Powell, Jayne Green, Roshan Lal, Vamsi Velchuru, Kamal Aryal, Muhammad Radzi Bin Abu Hassan, Goh Tiong Meng, Janisha Suriakant Patel, Shameera Pharveen Mohamed Gani, Chun Ren Lim, Thomas Guiel, Alexandre Akoulitchev, Dmitri Pchejetski

**Affiliations:** 1 Oxford BioDynamics Plc., Oxford OX4 2WB, UKalexandre.akoulitchev@oxfordbiodynamics.com (A.A.); 2 Norwich Medical School, University of East Anglia, Norwich NR4 7TJ, UK; 3 James Paget University Hospitals NHS Trust, Great Yarmouth NR31 6LA, UK; 4 Clinical Research Center, Hospital Sultanah Bahiyah, Jalan Langgar, Alor Setar 05460, Malaysia; 5 Island Hospital Penang, Jalan Macalister, George Town 10450, Malaysia; 6 Penang Reference Laboratory, Oxford BioDynamics Plc., Jalan Tanjung Tokong, George Town 10470, Malaysia; 7 Oxford BioDynamics Inc., Frederick, MD 21703, USA

**Keywords:** colorectal cancer, colonic polyp, chromosome conformations, epigenetics, blood-based biomarkers, 3D-genomic profiling, cancer diagnosis

## Abstract

Background/Objectives: Colorectal cancer (CRC) arises from the epithelial lining of the colon or rectum, often following a progression from benign adenomatous polyps to malignant carcinoma. Screening modalities such as colonoscopy, faecal immunochemical tests (FIT), and FIT-DNA are critical for early detection and prevention, but non-invasive methods lack sensitivity to polyps and early CRC. Chromosome conformations (CCs) are potent epigenetic regulators of gene expression. We have previously developed an epigenetic assay, EpiSwitch^®®^, that employs an algorithmic-based CCs analysis. Using EpiSwitch^®®^ technology, we have shown the presence of cancer-specific CCs in peripheral blood mononuclear cells (PBMCs) and primary tumours of patients with melanoma and prostate cancer. EpiSwitch^®®^-based commercial tests are now available to diagnose prostate cancer with 94% accuracy (PSE test) and response to immune checkpoint inhibitors across 14 cancers with 85% accuracy (CiRT test). Methods/Results/Conclusions: Using blood samples collected from *n* = 171 patients with CRC, *n* = 44 patients with colorectal polyps and *n* = 110 patients with a ‘clear’ colonoscopy we performed whole Genome DNA screening for CCs correlating to CRC diagnosis. Our findings suggest the presence of two eight-marker CC signatures (EpiSwitch^®®^ NST) in whole blood that allow diagnosis of CRC and precancerous polyps, respectively. Independent validation cohort testing demonstrated high accuracy in identifying colorectal polyps and early versus late stages of CRC with an exceptionally high sensitivity of 79–90% and a high positive prediction value of 60–84%. Linking the top diagnostic CCs to nearby genes, we have built pathways maps that likely underline processes contributing to the pathology of polyp and CRC progression, including TGFβ, cMYC, Rho GTPase, ROS, TNFa/NFκB, and APC.

## Simple Summary

In this retrospective (with partial prospective collection) case–control pilot study, we have used full blood samples from patients with colorectal cancer (CRC), patients with colorectal polyps, and patients with a ‘clear’ colonoscopy, and performed whole Genome DNA screening for chromosome conformations (CCs) correlating to CRC or polyp diagnosis. Our findings suggest the presence of two eight-marker CC signatures in whole blood that allow rapid and cost-effective diagnosis of CRC and precancerous polyps, respectively. These signatures showed high sensitivity and positive predictive value for CRC and precancerous polyps’ detection.

## 1. Introduction

Globally, colorectal cancer (CRC) is the third most common cancer type, accounting for 10% of all cancer cases. There were 1.9 million new cases and 930,000 deaths from the disease in 2020 [1]. CRC arises from the epithelial lining of the colon or rectum, often following a progression from benign adenomatous polyps to malignant carcinoma driven by genetic mutations, epigenetic alterations, and chronic inflammation [2]. More than 80% of CRC arises from adenomatous polyps and outgrowths of the lining of the colon or rectum, which are usually asymptomatic [3]. Some of the inherited genetic disorders, such as familial adenomatous polyposis and hereditary non-polyposis colon cancer, can cause CRC and are responsible for circa 5% of all CRC cases. However, 75–95% of CRC cases occur in people with little or no genetic risk [4].

CRC diagnosis is usually performed via colonoscopy and biopsy. The most common form of CRC is adenocarcinoma, constituting between 95% and 98% of all cases. Imaging such as computed tomography (CT), magnetic resonance imaging (MRI), or positron emission tomography (PET) scans are used to identify local and distal spread and to plan the optimal surgical approach [5]. Treatments for CRC include surgery, radiation therapy, chemotherapy, and immuno-oncology therapy with checkpoint inhibitors [6]. Early CRC stages (1 and 2) are confined within the wall of the colon, and could be treated radically with a combined surgical and medical approach. Late stages (3 and 4) often spread widely, and are not curable. The individual likelihood of survival depends on how advanced the cancer is.

In this context, screening from the age of 45 (50 in the UK) for early detection of CRC is considered an effective measure for preventing and decreasing deaths from CRC [7]. Screening for this cancer is effective for both early detection and prevention, and allows for diagnosis 2–3 years before the symptoms arise. Polyps often can be removed at the precancerous stage, and an effective screening programme has the potential to reduce CRC deaths by 60% [8]. Currently, the primary screening tests include colonoscopy, faecal occult blood testing (FOBT), and monitoring of cell-free DNA from CRC tumours in blood. Colonoscopy is considered a gold-standard diagnostic test for CRC, and its sensitivity is ~95%. However, up to 20% of colonoscopies are unsuccessful due to poor preparation or difficult anatomy and cancers in these patients are missed.

Furthermore, colonoscopy bears a significant risk of bleeding and bowel perforation—up to 4% each [9]. Two main non-invasive screening tests include guaiac-based or immunochemical-based detection of blood in stool, FOBT and faecal immunochemical test (FIT), respectively. They have high specificity and negative predictive value (NPV), but lower sensitivity and positive predictive value (PPV). The latest studies confirm that these tests may miss more than half of bowel cancer cases, especially at the lower stages [10]. Other screening options include virtual CT-colonoscopy and stool DNA screening testing (FIT-DNA). Colonoscopy via a CT scan is expensive, associated with radiation exposure, and cannot remove any detected abnormal growths as standard colonoscopy can. Stool FIT-DNA screening test also looks for altered DNA associated with CRC and precancerous lesions, but has a high level of false positive results [9]. The UK bowel cancer screening programme includes an FOBT test every two years between the ages of 50 and 74. FOBT overdiagnosis ranges from 2.0% to 7.6%, leading to unnecessary colonoscopies (with or without biopsies), patient distress, and extra costs [11].

With the advent of epigenetic research, it has become evident that epigenetic modifications like aberrant DNA methylation [12] and histone acetylation [13] are related to CRC onset. Three-dimensional chromatin conformations (CCs), as part of genomic regulatory architecture, are also potent epigenetic regulators of gene expression and cellular pathological phenotypes [14]. Long-range epigenetic alterations in CCs were found in primary CRC and circulating DNA from CRC patients [15].

**Figure 1:**
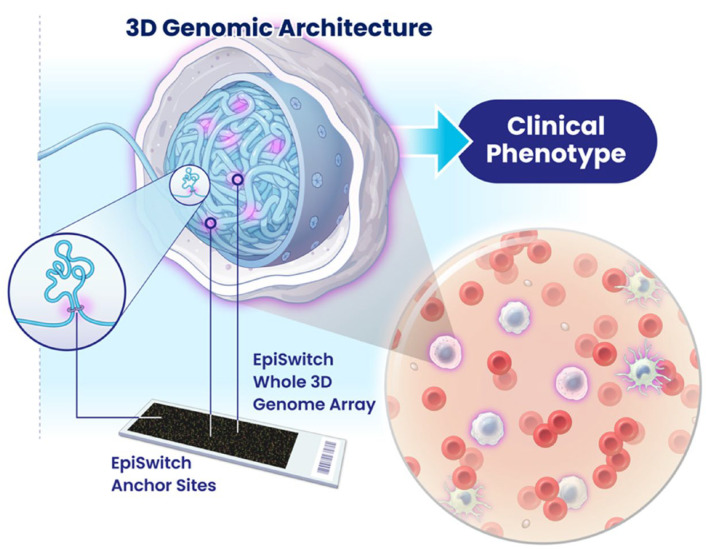
The principle of EpiSwitch^®®^ assay. DNA is isolated from peripheral blood mononuclear cells (PBMCs), is cross-linked using formaldehyde, digested, and ligated with the preference of cross-linked fragments. New sequences are formed where chromatin loops have been. These new sequences are predicted via relevance machine vector algorithm. Specific primers to these sequences are synthesised and placed on the DNA microarray, which detects whether the loop was present or not. Resulting markers are analysed using multivariate analysis yielding specific epigenetic signatures for selected patient cohorts [17,18,19,20,21].

We have previously developed an epigenetic assay, EpiSwitch^®®^ [16] (Figure 1), that employs an algorithmic-based CCs analysis. Using EpiSwitch^®®^ technology, we have shown the presence of cancer-specific CCs in peripheral blood mononuclear cells (PBMCs) and primary tumours of patients with melanoma [17,18] and prostate cancer [19]. In light of the regulatory role lately attributed to systemic exosome traffic, we have used indirect co-culture experiments or conditioned media, and demonstrated horizontal transfer of CCs between cultured cancer cells and monocytes without direct contact [20]. EpiSwitch^®®^-based commercial tests are now available to diagnose prostate cancer with 94% accuracy (PSE test) [21], and response to immune checkpoint inhibitors across 14 cancers with 85% accuracy (CiRT test) [22]. Interestingly, although the anchor sites associated with 3D genomic loops are scattered throughout genomes, by linking the top prognostic biomarkers to nearby genes (within 3 kb), it is possible to learn a great deal about the underlying processes contributing to the pathology of a disease and identify potential therapeutic strategies.

In this retrospective (with partial prospective collection) case–control study, we have used *n* = 325 full blood samples collected from *n* = 171 patients with CRC, *n* = 44 patients with colorectal polyps, and *n* = 110 patients with a ‘clear’ colonoscopy attending colorectal clinics, and performed whole Genome DNA screening for CCs correlating to CRC diagnosis. Our findings suggest the presence of two eight-marker CC signatures in whole blood that allow rapid and cost-effective diagnosis of CRC and precancerous polyps, respectively.

## 2. Materials and Methods

### 2.1. Patient Characteristics

In this retrospective case–control study with partial prospective recruitment, *n* = 325 whole blood samples (*n* = 110 controls (no polyp or cancer on colonoscopy), *n* = 44 polyp, and *n* = 171 CRC) were obtained from patients attending colorectal clinics at James Paget University Hospital, UK, Hospital Sultanah Bahiyah, Malaysia, and Island Hospital, Malaysia (Table 1). Inclusion criteria: clinical and histopathological diagnosis of CRC, precancerous lesion and normal colonoscopy, no prior history of any cancer, treatment naïve, and age range 18–75. A blood sample was taken prior to treatment. *n* = 225 samples (*n* = 68 control patients and *n* = 157 CRC) were collected retrospectively, and *n* = 100 patients were recruited through a prospective observational study yielding (*n* = 42 controls, *n* = 44 polyps, *n* = 14 CRC).

All samples were collected at the time of diagnosis and randomly allocated for training and test cohorts. The study was approved by the UK National Research Ethics Committee and Medical Research, as well as Ethics Committee Ministry of Health Malaysia, and conducted in accordance with Good Clinical Practice guidelines and the Declaration of Helsinki. All participants provided written informed consent. All data were pseudo-anonymised. All procedures and protocols were performed in accordance with the relevant guidelines and regulations.

### 2.2. Preparation of 3D Genomic Templates

A 5 mL full blood sample was collected from cancer patients and controls using BD Vacutainer^®®^ plastic EDTA tubes (Becton Dickinson, Wokingham, UK). The tubes were frozen and stored at −80 °C. Isolation of DNA from the whole cell lysate was performed as previously described, and DNA was fixed with formaldehyde. To identify interchromatin loops, fixed chromatin was digested into fragments with TaqI restriction enzyme, and the resulting DNA strands were joined, favouring cross-linked fragments. The cross-links were reversed, and PCR was performed using the primers designed using the algorithms of the EpiSwitch^®®^ software (as described in detail in [17,18,19,23]).

The 3C libraries were quantified using the Quant-iTTM Picogreen dsDNA Assay kit (Invitrogen, Paisley, UK) and normalised to 5 ng/μL prior to interrogation by PCR. The EpiSwitch^®®^ Explorer arrays were performed as previously published, with the modification of only one sample being hybridised to each array slide in the Cy3 channel. EpiSwitch^®®^ Explorer arrays, based on the Agilent SureSelect array platform, allow for the highly reproducible, non-biased interrogation of ~1.1 million anchor sites for 3D genomic interactions (964,631 experimental probes and 2500 control probes).

### 2.3. Custom Microarray Design

Custom microarrays were designed through the EpiSwitch^®®^ software that uses a pattern recognition algorithm based on DNA sequence, which operates on Bayesian modelling and yields a probability score of whether a region is involved in long-range chromatin interactions. The GRCh38 human genome assembly was annotated across ~1.1 million sites, and the potential to form long-range chromosome conformations [18,19,23,24,25,26]. The most probable interactions were identified and filtered on probabilistic score and proximity to protein, long non-coding RNA, or microRNA coding sequences. Predicted interactions were limited to EpiSwitch^®®^ sites larger than 10 kb and less than 300 kb apart. Repeat masking and sequence analysis were used to ensure unique marker sequences for each interaction. The EpiSwitch^®®^ Explorer array (Agilent Technologies, St. Clara, CA, USA, Product Code X-HS-AC-02), containing 60-mer oligonucleotide probes, was designed to interrogate potential 3D genomic interactions. In total, 964,631 experimental and 2500 control probes were added to a 1 × 1 M CGH microarray slide design. The experimental probes were placed on the design in singlicate with the controls in groups of 250. The control probes consisted of six different EpiSwitch^®®^ interactions generated during the extraction processes and used to monitor library quality. Four external inline control probe designs were added to detect non-human (*Arabidopsis thaliana*) spike-in DNA during the sample labelling protocol to provide a standard curve and control for labelling. The external spike DNA consists of 400 bp ssDNA fragments from genomic regions of *A. thaliana*. Array-based comparisons were performed as described previously, with the modification of only one sample being hybridised to each array slide in the Cy3 channel [18,19,23,24,25,26].

### 2.4. Microarray Statistical Analysis

Microarray readouts were normalised by background correction and quantile normalisation using the EpiSwitch^®®^ R analytic package, which is built on the Limma and dplyr libraries. Data were corrected for batch effects using the ComBat R script. Parametric (Limma R library, Linear Regression) and non-parametric (EpiSwitch^®®^ RankProd R library) statistical methods were performed to identify 3D genomic changes that demonstrated a difference in abundance between cancers and controls. The resulting data from both procedures were further filtered based on adjusted *p*-value (false discovery rate (FDR) correction) and abundance scores (AS). Only 3D genomic markers with adjusted *p*-value ≤ 0.05 and AS ≤−1.1 or ≥1.1 were selected. Both filtered lists from Limma and RankProd analysis were compared, and the intersection of the two lists was chosen for further processing.

#### 2.4.1. Step 1

Probes were selected based on their corrected *p*-value FDR, which is the product of a modified linear regression model. Probes below a *p*-value ≤ 0.1 were selected and then further reduced by their fold change (FC); probes’ FC have to be ≤−1.1 or ≥1.1 to be chosen for further analysis. The last filter was a coefficient of variation (CV); probes must be below ≤0.3.

#### 2.4.2. Step 2

The top 250 markers from the statistical lists were selected based on their FC for selection as markers for PCR translation.

#### 2.4.3. Step 3

The resultant markers from step 1, the statistically significant probes, form the basis of enrichment analysis using hypergeometric enrichment (HE). This analysis enables marker reduction from the significant probe list and, along with the markers from step 2, forms the list of probes translated onto the EpiSwitch™ PCR platform.

The statistical probes are processed by HE to determine which genetic locations have an enrichment of statistically significant probes, indicating which genetic locations are hubs of epigenetic difference.

The most significant enriched loci based on a corrected *p*-value are selected for probe list generation. Genetic locations below a *p*-value of 0.3 or 0.2 are selected. The statistical probes mapping these genetic locations, with the markers from step 2, form the high-value markers for EpiSwitch™ PCR translation.

### 2.5. Translation of Array-Based 3D Genomic Markers to PCR Readouts

In the discovery cohort, we analysed the leading array-derived markers using Oxford BioDynamics (OBD’s) proprietary primer design software. This process aimed to pinpoint genomic locations that are appropriate for a hydrolysis probe-based real-time PCR assay [27]. Briefly, the top array-derived markers associated with diagnostic potential were filtered on fold change and adjusted *p*-value. PCR primer probes were ordered from Eurofins genomics as salt-free primers. The probes were designed with a 5′ FAM fluorophore, 3′ IABkFQ quencher, and an additional internal ZEN quencher and ordered from Integrated DNA Technologies (iDT) [28]. Each assay was optimised using a temperature gradient PCR with an annealing temperature range from 58 to 68 °C. Individual PCR assays were tested across the temperature gradient alongside negative controls, including soluble and unstructured commercial TaqMan human genomic DNA control (Life Technologies, Thermo Fisher Scientific, Waltham, MA, USA), and a TE buffer-only negative control was used. Assay performance was assessed based on Cq values, the reliability of detection, and efficiency based on the slope of the individual amplification curves. Assays that passed the quality criteria and presented reliable detection differences between cancers and controls were used to screen individual patient samples.

### 2.6. EpiSwitch^®®^ PCR

Each patient sample was interrogated using triplicate real-time PCR. Each reaction consisted of 50 ng of EpiSwitch^®®^ library template, 250 mM of each of the primers, 200 mM of the hydrolysis probe, and a final 1X Kapa Probe Force Universal (Roche, Basel, Switzerland) concentration in a final 25 μL volume. The PCR cycling and data collection were performed using a CFX96 Touch Real-Time PCR detection system (Bio-Rad, Hercules, CA, USA). The annealing temperature of each assay was changed to the optimum temperature identified in the temperature gradients performed during translation for each assay. Otherwise, the same cycling conditions were used: 98 °C for 3 min, followed by 45 cycles of 95 °C for 10 s and 20 s at the identified optimum annealing temperature. The individual well Cq values were exported from the CFX manager software 3.1 after baseline and threshold value checks.

### 2.7. PCR Statistical Analysis

The 250 markers screened on 40 individual patient samples were subject to permutated logistic modelling with bootstrapping for 500 data splits and non-parametric Rank Product analysis (EpiSwitch^®®^ RankProd R library). Two machine learning procedures (Extreme Gradient Boosting: XGBoost and CatBoost) were used to reduce the feature pool further and identify the most predictive/prognostic 3D genomic markers. The resulting markers were then used to build the final classifying models using CatBoost and XGBoost. All analyses were performed using R statistical language with Caret, XGBoost, SHAPforxgboost, and CatBoost libraries (previously described in detail in [17,18,19,20,21]).

### 2.8. Biological Network/Pathway Analysis

Network analysis for functional/biological relevance of the 3D genomic markers was performed using the Hallmark Gene Sets and BioCarta and Reactome Canonical Pathway gene sets from the Molecular Signatures Database (MSigDB) [29]. Protein interaction networks were generated using the Search Tool for the Retrieval of Interacting Proteins (STRING) [30].

## 3. Results

In this retrospective (with partial prospective collection) case–control study, *n* = 325 whole blood samples (*n* = 110 controls, *n* = 44 polyps and *n* = 171 cancers) were obtained from patients attending colorectal clinics (Table 1). Patients were separated according to diagnosis (CRC, polyp, and control), and blood samples were taken prior to treatment. All control subjects had a ‘clear’ colonoscopy. Pre-lesion polyps were confirmed by a biopsy and histopathology. Pre-lesion and control samples were collected through a prospective observational study.

### 3.1. Microarrays

To design custom microarrays, we used the EpiSwitch^®®^ pattern recognition algorithm, which predicts long-range chromatin interactions through Bayesian modelling and provides a probabilistic score for each region. A total of ~1.1 million sites across the whole GRCh38 human genome assembly were identified as having the potential to form long-range chromosome conformations [18,19,23,24,25,26]. The most probable interactions were identified and filtered on probabilistic score and proximity to protein, long non-coding RNA, or microRNA coding sequences. Predicted interactions were limited to EpiSwitch^®®^ sites that were more significant than 10 kb and less than 300 kb apart. Repeat masking and sequence analysis were used to ensure unique marker sequences for each interaction.

**Figure 2:**
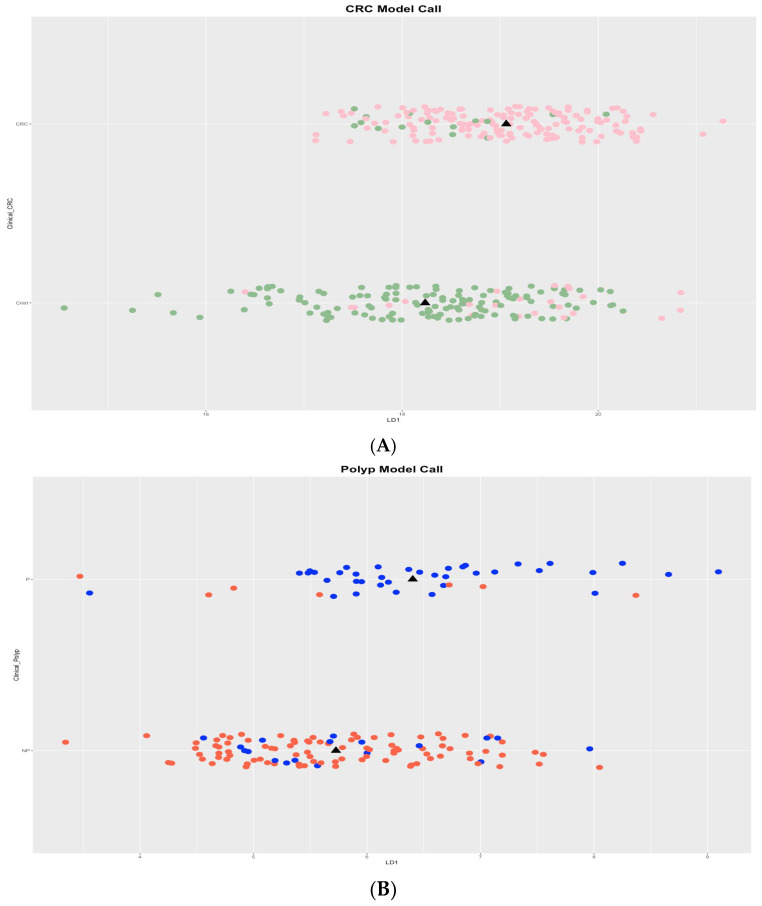
LDA plot of all CRC patients vs. control (**A**) and control vs. polyp (**B**). (**A**) The LDA plot of patients with CRC (pink circles) vs. control (green circles); the *y*-axis represents the two classes, while the *x*-axis is the LDA score. (**B**) The LDA plot of patients with polyp (blue circles) vs. control (orange circles). Analysis is based on whole genome profiling of all 964,631 3D genomic markers screened, without any marker reduction. Black triangle—median.

Whole-genome EpiSwitch^®®^ Explorer arrays were used to screen PBMCs samples collected at the time of confirmed diagnosis. Using Linear Discriminant Analysis (LDA), we showed a clear separation in all cohorts between CRC and control samples, as well as separation between polyp and non-polyp samples. This was performed prior to pre-selection or reduction of the 964,631 array markers (Figure 2A,B), suggesting that 3D genomic profiles associated with different clinical outcomes exist and can be distinguished.

**Figure 3:**
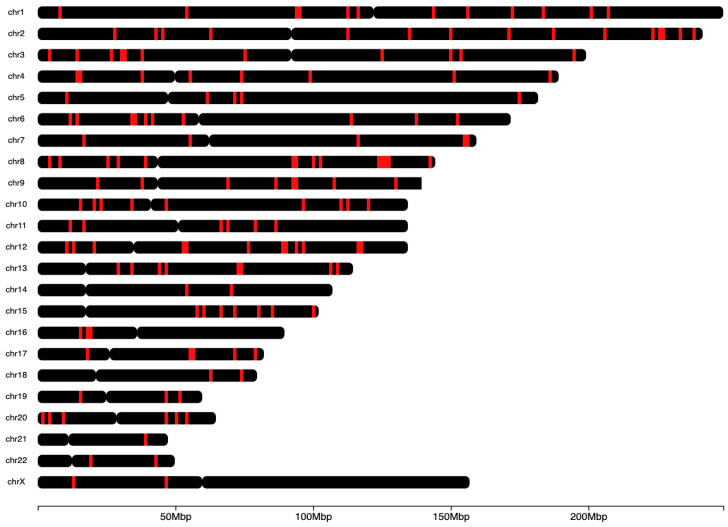
Genome-wide mapping of 250 3D genomic loci associated with CRC. Genomic locations and distribution of the top 250 3D genomic markers for CRC. Individual human chromosomes are shown on the *y*-axis (chr1-chr22 and the X chromosome). The heatmap shows the number of markers within a 0.3 Mb genomic window, with black representing a low density of markers and red indicating a high density of markers.

To evaluate the biological relevance of the observed separation of patients with CRC, polyps, and control patients, the 964,631 3D genomic markers from each patient were subject to statistical testing using both parametric testing (Limma) and non-parametric testing (EpiSwitch^®®^ RankProd), both procedures that correct for multiple testing by using FDR corrections. The RankProd approach also has a resampling step to control for random rank importance, adding another layer of statistical stringency in marker selection when testing many possibilities. The selected markers were filtered based on an adjusted FDR *p*-value ≤ 0.05 and high abundance scores (AS), ≤−1.1 or ≥1.1. Similar approaches and thresholds for FDR cut-offs have been used in previously published biomarker development studies [18,19,23,24,25,26,27]. Thus, we started with the 964,631 whole genome screened *cis*-interactions. After statistical filtering, the 250 3D genomic markers with the highest and lowest abundance scores were chosen for further analysis and PCR translation. The top 250 EpiSwitch^®®^ array markers were identified as statistically significant and consistently present, based on standard statistical analysis based on *p*-value and adjusted *p*-value, when screened on blood from patients with CRC, polyps, or control (Figure 2; a complete list can be found in Supplementary Table S1). These top 250 markers were randomly distributed throughout the human genome (Figure 3).

### 3.2. qPCR Validation of Biomarkers

To translate the EpiSwitch^®®^ Explorer array markers to a PCR-detectable assay for clinical use, primers to detect individual 3D genomic markers were generated and validated. Starting with whole blood samples from the training set, we identified feature reduction using machine learning methods on the initial pool of 250 3D genomic biomarkers through feature reduction of 12 markers with predictive power to differentiate between CRC patients and controls. These 12 array markers had strong identifiers for CRC and polyps, and were selected for qPCR translation.

The 12 qPCR markers were further refined on the training cohort of *n* = 74 CRC (both early and late stages) and non-CRC (polyps + controls), and the top eight markers from twelve were built into a classifier model using machine learning package XGBoost (Table 2). In a blind validation study of 251 samples, this marker set (EpiSwitch^®®^ no stool test (NST)) correctly classified 125 samples as CRC and 89 as non-CRC, with the remaining 37 samples as false positives or false negatives. It demonstrated high accuracy of 85%, 90% sensitivity, 79% specificity, and 84% PPV in stratifying patients with and without CRC (Table 3).

To assess the validity of this marker set for early-stage CRC detection, late-stage (stages 3 and 4) CRCs were excluded from analysis. In the resulting early-stage cohort of *n* = 149 samples, *n* = 31 samples were correctly classified as cancer (CRC), and *n* = 89 samples were correctly classified as non-cancer, with the remaining 29 samples being either false positives or negatives (Table 4). The test showed an accuracy of 81%, with 84% sensitivity, 79% specificity, 57% PPV, and 94% NPV in identifying patients with early stages of CRC.

Non-invasive detection of precancerous polyps remains a significant clinical challenge. While not technically a cancer, they often have precancerous features and have the potential to develop into CRC. Currently, only colonoscopy has good diagnostic accuracy, while existing non-invasive tests cannot be used for this purpose [31]. For patients classified as non-CRC, a second assessment has been evaluated. Based on the data from the polyps training cohort (*n* = 29), a separate set of top eight markers (with four markers overlapping with the CRC eight-marker set (Supplementary Table S2)) from twelve were built into a classifier model using the machine learning approach XGBoost (Table 5).

Using this new polyp-specific eight-marker set (EpiSwitch^®®^ NST), we have performed blinded validation on *n* = 142 non-CRC samples. Of those, 27 samples were correctly classified as polyp, and 90 were correctly classified as control. The remaining 25 samples are either false positives or negatives. This test achieved a high overall diagnostic accuracy of 82%, with 79% sensitivity, 83% specificity, 60% PPV, and 92% NPV to detect the presence of adenomas/precancerous lesions/polyps (Table 6).

Previous analysis has indicated that changes in the 3D chromosome architecture captured using EpiSwitch^®®^ biomarkers are also reflected in the broader region surrounding each biomarker. Analysis of these regions can give insights into the causes of the observed phenotype [18,19,23,24,25,26,27]. The genomic positions of the 250 3D genomic markers were mapped to enable the identification of the three closest protein-coding loci. Potential functional roles for these loci were obtained using Hallmark Gene Sets, BioCarta, and Reactome canonical pathway analysis. Pathway analysis for the eight colorectal classifier markers showed multiple pathways involved in CRC, including transforming growth factor beta (TGFβ), cMYC, Rho GTPase, reactive oxygen species (ROS), and adenomatous polyposis coli (APC) (Supplementary Table S3). A similar analysis of the eight-marker polyp classifier showed pathways related to TGFβ, PAX8, epithelial–mesenchymal transition (EMT), tumour necrosis factor alfa (TNFα)/nuclear factor kappa B (NFκB), ROS, and APC (Supplementary Table S4).

**Figure 4:**
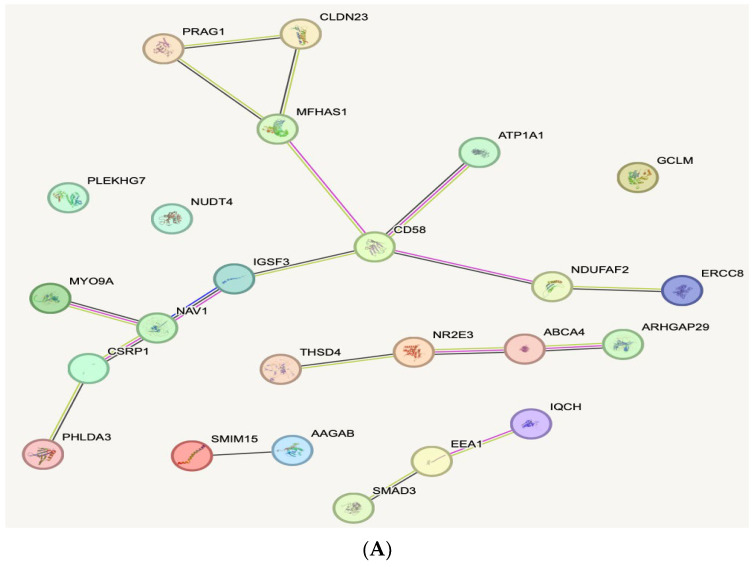

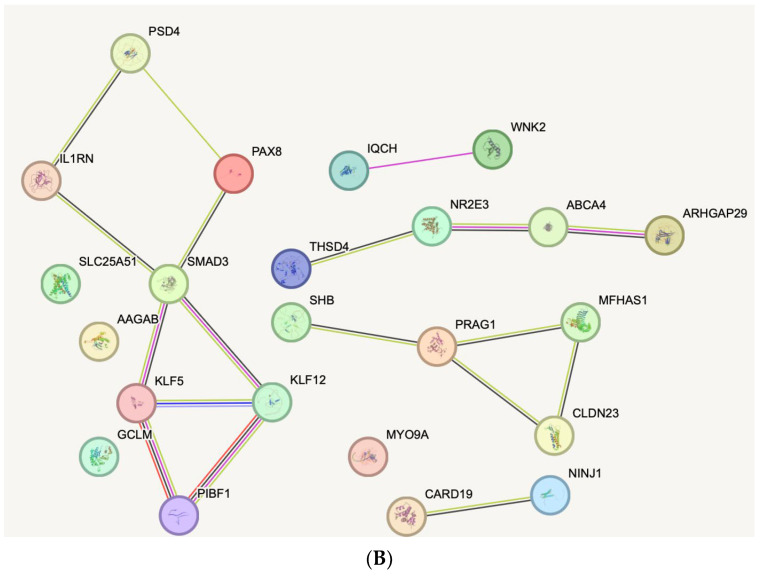
STRING network of the eight colorectal classifier markers (**A**) and eight polyp classifier markers (**B**). STRING Network associated with CRC and colorectal polyps. The proteins encoded by genes in the vicinity of the top 3D genomic markers related to CRC and polyps reveal a network with hubs, as shown.

When evaluating the biological function of the genes within the genomic regions identified as being dysregulated between patients with CRC, polyps and controls, several biological pathways with known associations with cancer were identified. Analysis of the top 3D genomic markers associated with CRC using the Search Tool for Retrieval of Interacting Genes (STRING) database revealed eight marker CRC and polyp panels protein–protein interaction networks with hubs on cluster of differentiation (CD)58, mothers against decapentaplegic homologue (SMAD)3, and interleukin 1 receptor (IL1R) (Figure 4A,B). The interaction scores in STRING do not represent the strength or specificity of a given interaction, but instead are meant to express approximate confidence, on a scale of zero to one, of the association being true, given all the available evidence. In this analysis we used low confidence to generate the network ≤ 0.15, with all other settings set to default.

## 4. Discussion

One of the main challenges in CRC management is reliable early detection, with active treatments and prophylactics of precancerous lesions offering effective cures and reduced mortality. Colonoscopy with subsequent biopsy remains a gold-standard test for all types of CRC and precancerous lesions. However, it is invasive, costly, and requires secondary care settings and expertise. Most current early detection tests in CRC and polyps perform poorly in detecting the early stages of CRC, resulting in significant false positive readings. Most precancerous polyps are missed (Table 7) [32,33,34,35].

Epigenetics is the study of heritable changes in phenotype that do not involve alterations in the DNA sequence [36]. These changes are powered by the modification of gene expression, and occur through four principal mechanisms: DNA methylation, histone modification, changes in 3D chromosome structures, and chromosomal looping [36]. Chromosomal loops are the dynamic 3D chromatin structures that exist in all cells and determine gene expression in so-called “active cluster” regions. They are potent regulators of gene expression during tumorigenesis [37].

Genome-wide Association Studies (GWAS) have shown that epigenetic alterations are often more important than genetic mutations during oncogenic transformation. For example, in cancers, loss of gene expression occurs about ten times more frequently by epigenetic transcription silencing than by mutations [38]. One interesting observation from GWAS was that most allele regions of the genome that confer risk to cancer are outside of known protein-coding regions [39]. In CRC, only ~5% of all cases are due to inherited genetic disorders (such as familial adenomatous polyposis), and 75–95% of CRC cases occur in people with little or no genetic risk [4]. There is a growing understanding of the role of epigenetic regulation in CRC progression (reviewed in [40]).

Chromatin conformations are often controlled by non-coding RNAs, which may also regulate tumour-specific conformations [41]. It was shown that tumour cells can secrete non-coding RNAs endocytosed by neighbouring or circulating cells and change their chromosomal conformations in a process called “horizontal transfer” [42,43].

We have performed a proof-of-concept study where we co-cultured prostate cancer cells and primary macrophages in a Boyden chamber (through a membrane of 0.45 µM without direct contact) or exposed macrophages to prostate cancer cell-conditioned media. In both cases, we have detected new prostate cancer-specific chromosomal conformation changes in the macrophages [20]. We have confirmed these findings in patients with melanoma, where we have shown the presence of identical melanoma-specific chromatin conformations in primary tumours of melanoma patients and their PBMCs [17,18]. Blood cell fractionation showed that the detected signature comes from PBMCs, not circulating tumour cells [17]. A similar approach was used to confirm the presence of prostate cancer-specific signatures in PBMCs from prostate cancer patients.

Using our EpiSwitch^®®^ technology, we have created a new diagnostic test for melanoma [17,18], thyroid cancer [26], and prostate cancer [19,21] (PSE test), capable of identifying prostate cancer presence with 94% overall accuracy. This test is currently available in the clinic.

In this retrospective (with partial prospective collection) case–control study, we have analysed *n* = 325 whole blood samples from *n* = 171 patients with CRC, *n* = 44 patients with colorectal polyps, and *n* = 110 patients with a ‘clear’ colonoscopy.

Initially, all three cohorts were compared using the whole-genome EpiSwitch^®®^ Explorer arrays, which showed a remarkable separation between CRC and non-cancer, and polyp and control, without pre-selection or reduction of the 964,631 array markers (Figure 2A,B). This aligns with our previous findings in prostate cancer [19,21] and melanoma [17,18], where CCs profiles could accurately distinguish cancer patients from controls. Biomarker reduction and translation from DNA CHIPs to PCR is a crucial step in EpiSwitch^®®^ technology, where statistical filtering and machine learning methods allowed us to initially reduce to 250 and then to 12 3D genomic biomarkers.

Unlike other cancers, CRC often has a well-identifiable precancerous stage manifesting as colonic polyps. These polyps could be relatively easily found during colonoscopy, but other non-invasive methods have difficulties identifying them (Table 7). Since polyps are usually asymptomatic and can be easily removed (therefore reducing the cancer risk), their early non-invasive identification is the next challenge for CRC management. Bearing this in mind, we have used the machine learning package XGBoost to train our classifier system further to detect CRC and polyps separately. This resulted in establishing two independent but overlapping eight-marker signatures for a dual purpose (EpiSwitch^®®^ NST) based on twelve markers (Table 2 and Table 5). The first eight-marker signature, when tested in a blind validation study of 251 samples, showed 90% sensitivity, 79% specificity, and 84% PPV in identifying all CRC (Table 3), and 84% sensitivity, 79% specificity, and 57% PPV in identifying patients, with stages 1 and 2 of CRC (Table 4). This is a remarkable finding, as most non-invasive tests, while having high specificity and NPV, have low sensitivity and PPV, resulting in a large number of both missed diagnoses and false positive diagnoses, causing anguish and unnecessary procedures. Similarly, for polyp detection, a second eight-marker signature (Table 5) (EpiSwitch^®®^ NST) showed 79% sensitivity, 83% specificity, and 60% PPV to detect the presence of adenomas/precancerous lesions/polyps. This is a remarkable accuracy compared to available analogues (Table 7). Thus, using a 12-marker set of EpiSwitch^®®^ NST could offer a consecutive two-step stratification of high accuracy (>80%) in detecting early stages of CRC and precancerous lesions/polyps for non-CRC cases.

Interestingly, the two developed eight-marker sets had four common CCs (Supplementary Table S2), likely reflecting the pathophysiological relationship between precancerous lesions and CRC. SGK223 (PRAG1) is involved in Notch signalling and epithelial–mesenchymal transition (EMT) in CRC [44]. SMAD3 is a vital member of the TGFβ pathway (also a key player in EMT), and is deregulated in CRC [45].

The additional benefit of whole-genome EpiSwitch^®®^ Explorer arrays is the ability to map the genomic positions of identified CCs, enabling the identification of the three closest protein-coding loci. Potential functional roles for these loci were obtained using Hallmark Gene Sets, BioCarta, and Reactome canonical pathway analysis. Pathway analysis for the eight colorectal classifier markers showed several pathways involved in CRC, including TGFβ, cMYC, Rho GTPase, ROS, and APC (Supplementary Table S3). A similar analysis of the eight-marker polyp classifier showed TGFβ, PAX8, EMT, TNFα/NFκB, ROS, and APC (Supplementary Table S4). The pathway overlap is evident, and likely represents a possible pathway of CRC progression, emphasising EMT and inflammation. Notably, the aberrations in these pathways are already present in precancerous lesions. This may significantly facilitate their detection, as at that stage, they rarely present with any symptoms or physical manifestations (such as bleeding due to extensive growth or neovascularisation) as CRC lesions do. Analysis of the top 3D genomic markers associated with CRC using the STRING database revealed the eight-marker CRC and polyp panels’ protein–protein interaction networks with hubs on CD58, SMAD3, and IL1R (Figure 4A,B). These three key signalling molecules have a well-documented association with CRC. Interestingly, they are all simultaneously involved in two fundamental mechanisms: EMT and inflammation [46,47,48,49]. While the network analysis does not directly benefit the establishment and validation of a biomarker test, these identified pathways may suggest further targeting therapies that can be used at various stages of the disease, including possible earlier stages in the adjuvant or neoadjuvant settings.

## 5. Limitations of the Study

This is a biomarker discovery study with yes/no outcome to the presence of epigenetic CC markers capable of identifying CRC and precancerous polyps. The current study setup (retrospective (established diagnosis of control/cancer) + prospective (recruitment of screening patients)) allowed for a proportional balance between the control and cancer patients matching for sex and age, but introduced an imbalance between the number of control vs. cancer vs. polyp patients. Full matching of the patients in this study was impossible due to the numbers involved in the prospective cohort. A prospective cohort allowed an additional insight into the diagnosis of low-grade cancers and polyps. Importantly, test and validation cohorts were completely independent, allowing greater confidence in this study’s results. Further prospective matched studies based on real-world data should be planned to fully support these findings.

## 6. Conclusions

In this retrospective study using whole Genome DNA screening for CCs correlating to CRC and polyp diagnosis, we have identified two eight-marker CC signatures (EpiSwitch^®®^ NST) in whole blood that allow for rapid and cost-effective diagnosis of CRC and precancerous polyps, respectively. Both diagnostic sets demonstrate high sensitivity and PPV, which is particularly vital since existing blood and stool tests lack those attributes, especially in detection of pre-cancerous polyps and early-stage CRC. Genomic pathway analysis revealed signalling pathways related to the identified CCs, many of which have already been associated with CRC. Considering the significant signature/pathway overlap between polyps and CRC, these pathways are likely to play a key role in CRC pathophysiological progression, and may suggest further targeted therapies in CRC management.

## Figures and Tables

**Table 1:** Summary of clinical characteristics for patient cohorts used for biomarker discovery.

Cohort	N (Total)	Prospective	Retrospective	Male	Female	Age (Mean)
**Control**	110	42	68	56	54	61
**Polyp**	44	44	0	29	15	63
**CRC**	171	14	157	89	82	64

**Table 2:** Eight PCR biomarkers for CRC classifier.

qPCR Markers	Array Marker	Probe Sequence	*p*.Value	adj.*p*.Val	FC	Gene	GeneDist
obd156_q1177_q1179	ORF1_1_116481182_116484855_116627241_116630872_RF	TTGACATAGGACCTCAGCAGAGAGCAGCTCGAGATCCACCCACGTTGTTGCATGTATCAA	2.63 × 10^−2^	1.00 × 10^0^	−1.30	RP5-1086K13.1; CD58; NAP1L4P1; MIR548AC; IGSF3; AL355794.1; RP4-655J12.4; MIR320B1	0; 0; 0; 0; 0; 0; 2341; 40878
obd156_q1313_q1315	ORF1_5_61009121_61015983_61116919_61125541_FR	GAGGCAGGCAGATCACAAGGTCAAGAGATCGATAAGTACATGAGAAATAAACAAAATTCA	2.33 × 10^−7^	8.13 × 10^−5^	−1.39	NDUFAF2; CTC-436P18.4; ERCC8; CTC-436P18.5	0; 0; 64049; 20306
obd156_q1301_q1303	ORF1_12_93013996_93019448_93102345_93106201_FR	TGATGGACTTATGGACTCATTCACTGCATCGATATGGCTCATGCCATTTTATGTGCTATC	2.02 × 10^−8^	2.64 × 10^−5^	1.45	RP11-511B23.1; RP11-511B23.2; Y_RNA; RP11-511B23.4; RPL41P5; RP11-202G11.2; AC138123.1; RNU6-1329P; NACAP3	0; 0; 0; 0; 0; 0; 0; 61252; 17863
obd156_q1185_q1187	ORF1_1_201477609_201480715_201569360_201570965_RF	ACAAAGCTATCTCATTTCCTGAGCTTCATCGAGGTGAGGAGATCATGGATGAGTTTTTTA	2.47 × 10^−2^	1.00 × 10^0^	1.54	CSRP1; RP11-134G8.7; RP11-134G8.5; RP11-134G8.6; PHLDA3; NAV1	0; 0; 0; 0; 8373; 51921
obd156_q1245_q1247	ORF1_8_8307248_8309141_8529093_8530943_RF	CAATAATTCATTCTTCTTCATCAGTCCTTCGAACTCCTGACTCAGGAGATCTATCCACCT	1.62 × 10^−2^	1.00 × 10^0^	−1.34	SGK223; CTA-398F10.1; CTA-398F10.2; FAM86B3P; CTD-3023L14.3	0; 0; 0; 62384; 24796
obd156_q1217_q1219	ORF1_1_94060570_94064104_94081020_94084795_RF	TCTTGCCGGGAGTACTCTTCAAACTCCTTCGACATGATGGAGAAGCTGTCCAGGAACCAG	1.10 × 10^−6^	1.63 × 10^−4^	1.54	ABCA4; RP5-837O21.2; RP11-78O9.1	0; 125327; 60317
obd156_q1297_q1299	ORF1_15_71449255_71457687_71567140_71571578_RR	GTACTGAATAATAGTGTATGTGTTTATGTCGACTGTACTGGCGGACCCTATAAGAGGCAG	6.85 × 10^−6^	4.21 × 10^−4^	1.46	THSD4; RP11-1123I8.1; RP11-592N21.2; AC104938.1	0; 0; 100785; 201033
obd156_q1225_q1227	ORF1_15_67079527_67081854_67195948_67198335_RF	ATCTGTCCCAATCCTTTATCCTTCTAGCTCGAGTCAGCAGTGTTGACTGTTAGCAAATCA	1.80 × 10^−7^	7.03 × 10^−5^	1.65	SMAD3; RP11-342M21.2; RP11-798K3.2; AAGAB	0; 0; 20275; 2699

**Table 3:** Eight-marker diagnostic set validation cancer (all stages) versus non-cancer.

Test	Present	*n*	Absent	*n*	Total
**Yes**	**True positive**	**125**	**False positive**	**25**	**150**
**No**	**False negative**	**14**	**True negative**	**89**	**103**
**Total**		**139**		**114**	
**Statistic**	**Value (%)**		**95% Cl**		
Sensitivity	89.93		83.68 to 94.38		
Specificity	79.46		70.80 to 86.51		
Positive Likelihood Ratio	4.38		3.03 to 6.33		
Negative Likelihood Ratio	0.13		0.08 to 0.21		
Disease prevalence	55.38		49.00 to 61.63		
Positive Predictive Value	84.46		78.99 to 88.71		
Negative Predictive value	86.41		79.31 to 91.33		
Accuracy	85.26		80.26 to 89.40		

**Table 4:** Eight-marker diagnostic set validation early cancer (stages 1 and 2) versus non-cancer.

Test	Present	*n*	Absent	*n*	Total
**Yes**	**True positive**	**31**	**False positive**	**23**	**54**
**No**	**False negative**	**6**	**True negative**	**89**	**95**
**Total**		**37**		**112**	
**Statistic**		**Value (%)**		**95% Cl**	
Sensitivity		83.78		67.99 to 93.81	
Specificity		79.46		70.80 to 86.51	
Positive Likelihood Ratio		4.08		2.76 to 6.03	
Negative Likelihood Ratio		0.2		0.10 to 0.43	
Disease prevalence		24.83		18.13 to 32.57	
Positive Predictive Value		57.41		47.69 to 66.58	
Negative Predictive value		93.68		87.64 to 96.88	
Accuracy		80.54		73.26 to 86.56	

**Table 5:** Eight PCR biomarkers for polyps classifier.

qPCR Markers	Array Marker	Probe Sequence	*p*.Value	adj.*p*.Val	FC	Gene	GeneDist
obd156_q1205_q1207	ORF1_13_73435053_73437099_73484222_73486544_RF	ACACACAGTAGGTAATTAATACGGTGGATCGAAGTACGCTCTAGTTATACGAGGCTTGTT	4.43 × 10^−8^	3.46 × 10^−5^	1.42	LINC00393; MARK2P12; LINC00392	0; 26702; 77701
obd156_q1213_q1215	ORF1_9_37919925_37923489_38002100_38004773_FR	CCGAGGTCCCGAGACTATCTGCCAATCCTCGATTCTCTGGTTTTCCAGTTTGTCTATCTT	2.49 × 10^−7^	8.27 × 10^−5^	−1.37	RP11-613M10.9; SHB; RNU7-124P; SLC25A51; AL161448.1	0; 0; 0; 15573; 141722
obd156_q1273_q1275	ORF1_2_113209902_113215780_113275966_113277494_FR	CCAACACCACCCCAAATGCCGGGGCACGTCGAGCGTCCCCGGTTATTGGGAAGGGTGCGC	1.79 × 10^−2^	1.00 × 10^0^	−1.46	PAX8-AS1; PAX8; RP11-65I12.1; PSD4; IGKV1OR2-108	0; 0; 0; 507; 128903
obd156_q1293_q1295	ORF1_9_93218632_93223726_93274460_93278066_RF	TTTATATAACAATGTTTTTTTCAAGGCTTCGAGCAGACATTTCCCCGTCAGGAAGTAACA	1.12 × 10^−7^	5.53 × 10^−5^	−1.44	WNK2; RP11-370F5.4; C9orf129	0; 70077; 40134
obd156_q1245_q1247	ORF1_8_8307248_8309141_8529093_8530943_RF	CAATAATTCATTCTTCTTCATCAGTCCTTCGAACTCCTGACTCAGGAGATCTATCCACCT	1.62 × 10^−2^	1.00 × 10^0^	−1.34	SGK223; CTA-398F10.1; CTA-398F10.2; FAM86B3P; CTD-3023L14.3	0; 0; 0; 62384; 24796
obd156_q1217_q1219	ORF1_1_94060570_94064104_94081020_94084795_RF	TCTTGCCGGGAGTACTCTTCAAACTCCTTCGACATGATGGAGAAGCTGTCCAGGAACCAG	1.10 × 10^−6^	1.63 × 10^−4^	1.54	ABCA4; RP5-837O21.2; RP11-78O9.1	0; 125327; 60317
obd156_q1297_q1299	ORF1_15_71449255_71457687_71567140_71571578_RR	GTACTGAATAATAGTGTATGTGTTTATGTCGACTGTACTGGCGGACCCTATAAGAGGCAG	6.85 × 10^−6^	4.21 × 10^−4^	1.46	THSD4; RP11-1123I8.1; RP11-592N21.2; AC104938.1	0; 0; 100785; 201033
obd156_q1225_q1227	ORF1_15_67079527_67081854_67195948_67198335_RF	ATCTGTCCCAATCCTTTATCCTTCTAGCTCGAGTCAGCAGTGTTGACTGTTAGCAAATCA	1.80 × 10^−7^	7.03 × 10^−5^	1.65	SMAD3; RP11-342M21.2; RP11-798K3.2; AAGAB	0; 0; 20275; 2699

**Table 6:** Blinded validation of eight-marker ‘polyp’ diagnostic set for the presence of polyp versus control.

Test	Present	*n*	Absent	*n*	Total
**Yes**	**True positive**	**27**	**False positive**	**18**	**45**
**No**	**False negative**	**7**	**True negative**	**90**	**97**
**Total**		**34**		**108**	
**Statistic**		**Value (%)**		**95% Cl**	
Sensitivity		79.41		62.10 to 91.30	
Specificity		83.33		74.94 to 89.81	
Positive Likelihood Ratio		4.76		3.02 to 7.51	
Negative Likelihood Ratio		0.25		0.13 to 0.48	
Disease prevalence		23.94		17.19 to 31.82	
Positive Predictive Value		60		48.76 to 70.28	
Negative Predictive value		92.78		86.86 to 96.16	
Accuracy		82.39		75.12 to 88.27	

**Table 7:** Comparative efficacy of non-invasive diagnostic tests for CRC and polyps.

Detection of Precancerous Lesions (Polyps)						
	EpiSwitch^®®^ NST	Cologuard	FIT	Freenome PREEMPT CRC^®®^	Guardant Shield^®®^	Colonoscopy
**Sensitivity**	79%	43%	23%	13%	13%	75%
**Specificity**	83%	91%	95%	92%	90%	89%
**PPV**	60%	36%	35%	39%	17%	40%
**NPV**	93%	93%	91%	73%	86%	99%
**Accuracy**	82%	86%	87%	70%	77%	86%
**Detection of early-stage (I/II) CRC**						
	**EpiSwitch^®®^ NST**	**Cologuard**	**FIT**	**Freenome PREEMPT CRC^®®^**	**Guardant Shield^®®^**	**Colonoscopy**
**Sensitivity**	84%	90%	60%	79%	65%	75%
**Specificity**	79%	91%	95%	92%	90%	89%
**PPV**	57%	2%	3%	3%	1%	80%
**NPV**	94%	100%	100%	100%	100%	86%
**Accuracy**	81%	91%	95%	92%	90%	80%
**Detection of CRC (stages I–IV)**						
	**EpiSwitch^®®^ NST**	**Cologuard**	**FIT**	**Freenome PREEMPT CRC^®®^**	**Guardant Shield^®®^**	**Colonoscopy**
**Sensitivity**	90%	97%	71%	82%	83%	75%
**Specificity**	79%	91%	95%	92%	90%	89%
**PPV**	84%	2%	3%	3%	2%	80%
**NPV**	86%	100%	100%	100%	99%	86%
**Accuracy**	85%	91%	95%	92%	90%	80%

Abbreviations: positive predictive value, PPV; negative predictive value, NPV.

## Data Availability

The data presented in this study are available on request from the.corresponding author.

## Supplementary Materials

The following supporting information can be downloaded at: https://www.mdpi.com/article/10.3390/cancers17030521/s1, Table S1: Top 250 EpiSwitch array markers were identified as statistically significant and consistently present, based on standard statistical analysis based on P Value and adjusted P value, when screened on bloods from patients with cases of healthy, colorectal cancer and polypstitle; Table S2: Four common markers in SGK223, ABCA4, THSD4 and SMAD3 genes shared between CRC and polyp classifiers. Table S3: Pathway analysis for the eight colorectal classifier markers, the table shows top pathways from EpiSwitch 3D genome mapping app, the data is ranked by score (−log2 of hypergeometric *p*.value). Table S4: Pathway analysis for the 8 polyp classifier markers, the table shows top pathways from EpiSwitch 3D genome mapping app, the data is ranked by score (−log2 of hypergeometric *p*.value).
