# Supplementary material for: A New Blood-Based Epigenetic Diagnostic Biomarker Test (EpiSwitch®® NST) with High Sensitivity and Positive Predictive Value for Colorectal Cancer and Precancerous Polyps
Source: Cancers (Basel). 2025 Feb 4;17(3):521. doi: 10.3390/cancers17030521 (PMC11816175; doi:10.3390/cancers17030521)
Supplement: Supplementary file 1 [file cancers-17-00521-s001.zip › Suppl table S4 Pathway analysis for the 8 Polyp classifier markers.pdf]

**Supplementary Table S4. Pathway analysis for the 8 polyp classifier markers; the table shows top pathways from the EpiSwitch 3D genome mapping app, and the data is ranked by score (-log2 of hypergeometric *p*-value)**

| Pathway | PathwaySource | PathwaySize | Input_list | Overlap | Score | V1 | V2 | V3 | V4 | V5 | V6 | V7 |
|---|---|---|---|---|---|---|---|---|---|---|---|---|
| PAX8 Targets in Thyroid Dysgenesis (Hypothesis) | Elsevier | 15 | 24 | 2 | 12.774 | SMAD3 | PAX8 | | | | | |
| TGFB Signaling activation by Blocking of Tumor Suppressors | Elsevier | 74 | 24 | 2 | 8.16 | SMAD3 | KLF5 | | | | | |
| TGF-beta signaling pathway | KEGG | 95 | 24 | 2 | 7.459 | SMAD3 | THSD4 | | | | | |
| Colorectal Neoplasms | DisGeNet | 1073 | 24 | 5 | 7.297 | ABCA4 | GCLM | PAX8 | KLF5 | IL1RN | | |
| Bile Acid Metabolism | Hallmark/Age | 113 | 24 | 2 | 6.978 | ABCA4 | GCLM | | | | | |
| Colon (non-specific) lesion | DisGeNet | 7 | 24 | 1 | 6.904 | SMAD3 | | | | | | |
| Mesodermal.commitment.pathway | Wiki | 149 | 24 | 2 | 6.219 | SMAD3 | KLF5 | | | | | |
| Epithelial.to.mesenchymal.transition.in.colorectal.cancer | Wiki | 163 | 24 | 2 | 5.975 | SMAD3 | CLDN23 | | | | | |
| TNF-alpha Signaling via NF-kB | Hallmark/Age | 201 | 24 | 2 | 5.414 | NINJ1 | SMAD3 | | | | | |
| Colorectal Carcinoma | DisGeNet | 2931 | 24 | 7 | 5.244 | IL1RN | ABCA4 | GCLM | PAX8 | KLF5 | PSD4 | SMAD3 |
| GUCY2C Signaling in Colorectal Cancer | Elsevier | 27 | 24 | 1 | 4.974 | SMAD3 | | | | | | |
| Reactive Oxygen Species Pathway | Hallmark/Age | 50 | 24 | 1 | 4.106 | GCLM | | | | | | |
| Malignant tumor of colon | DisGeNet | 2001 | 24 | 5 | 4.039 | AAGAB | IL1RN | GCLM | KLF5 | SMAD3 | | |
| TGF-beta Signaling | Hallmark/Age | 55 | 24 | 1 | 3.973 | SMAD3 | | | | | | |
| Multiple polyps | DisGeNet | 62 | 24 | 1 | 3.806 | SMAD3 | | | | | | |
| Adenomatous Polyposis Coli | DisGeNet | 392 | 24 | 2 | 3.703 | AAGAB | SMAD3 | | | | | |
| Chromosomal.and.microsatellite.instability.in.colorectal.cancer | Wiki | 74 | 24 | 1 | 3.561 | SMAD3 | | | | | | |
| Colorectal Cancer | DisGeNet | 3298 | 24 | 6 | 3.155 | IL1RN | GCLM | ABCA4 | PAX8 | KLF5 | SMAD3 | |
| Metastatic Colorectal Cancer | Elsevier | 122 | 24 | 1 | 2.882 | SMAD3 | | | | | | |
| ActivatingInvasionMotility | Hallmark/Age | 1835 | 24 | 4 | 2.868 | IL1RN | NINJ1 | SMAD3 | PIBF1 | | | |
| Colon Carcinoma | DisGeNet | 2091 | 24 | 4 | 2.424 | AAGAB | IL1RN | KLF5 | SMAD3 | | | |
| Colonic Neoplasms | DisGeNet | 778 | 24 | 2 | 2.148 | IL1RN | SMAD3 | | | | | |
