# Supplementary material for: A New Blood-Based Epigenetic Diagnostic Biomarker Test (EpiSwitch®® NST) with High Sensitivity and Positive Predictive Value for Colorectal Cancer and Precancerous Polyps
Source: Cancers (Basel). 2025 Feb 4;17(3):521. doi: 10.3390/cancers17030521 (PMC11816175; doi:10.3390/cancers17030521)
Supplement: Supplementary file 1 [file cancers-17-00521-s001.zip › Supplementary table S1 Top 250 EpiSwitch array markers.pdf]

Supplementary Table S1. Top 250 EpiSwitch array markers were identified as statistically significant and consistently present, based on standard statistical analysis based on *p*-Value and adjusted *p*-value, when screened on bloods from healthy patients, and those with cases of colorectal cancer and polyps.

Array probe	P.Value	adj.P.Val	FC	Gene	GeneDist
ORF1_8_128145591_128153318_128187181_128189922_RF	5.23E-09	1.62211E-05	-1.57405338	MIR1208;FKSG59;RN7SKP226	0;27207;30583
ORF1_13_73346451_73348615_73424739_73428101_RF	1.93E-10	4.33234E-06	-1.532332118	RP11-309J13.1;MARK2P12;LINC00393;RNY1P8;LINC00392	0;0;0;119192;136144
ORF1_12_52177892_52180734_52255968_52258253_RR	4.91E-08	3.65469E-05	-1.520576628	KRT80;C12orf80;LINC00592;METTL7AP1;KRT7;AC021066.1;RP3-416H24.1;KRT86;KRT87P;AC078864.2;RP11-845M18.7	0;0;0;0;0;0;0;0;63881;16395
ORF1_3_149256688_149258162_149401952_149407044_FF	0.000000218	7.93193E-05	-1.513237992	CPHL1P;RP11-206M11.7;TM4SF18;RP11-2G17.1;TM4SF1;TM4SF1-AS1;RP11-278L15.2;CP;RP11-167H9.1	0;0;0;0;0;0;0;34634;26001
ORF1_21_38803260_38809151_38887013_38889232_FR	0.000000249	8.26688E-05	-1.51259886	ETS2;AP001042.1;AF064858.6;AP001043.1;LINC00114;SNORA62	0;0;0;0;55801;5554
ORF1_5_175273953_175275690_175377094_175381814_RF	1.14E-08	2.10181E-05	-1.502124372	ARL2BPP6;CTC-281M20.1;DRD1	0;278441;58226
ORF1_4_99304463_99307201_99400690_99404136_FR	8.66E-08	4.9976E-05	-1.501260337	ADH1B;ADH1C;RP11-696N14.1;ADH7	0;0;3108;8126
ORF1_3_74493927_74495965_74611524_74614255_RR	0.000000151	6.41881E-05	-1.495668629	CNTN3;AC128653.1;AKR1B1P2;NIPA2P2	0;0;357036;469901
ORF1_6_137004019_137005960_137148117_137153597_FF	2.53E-09	1.04444E-05	-1.488175665	IL20RA;IL22RA2;RP11-204P2.3;IFNGR1	0;0;4516;43888
ORF1_2_223614112_223615747_223705172_223708414_RR	0.00000204	0.000223528	-1.481020479	AC012512.1;SCG2;AP1S3	0;11610;43273
ORF1_11_86466088_86493462_86748000_86749113_FR	0.0000549	0.001343791	-1.472603648	RP11-317J19.1;ME3;CTD-2005H7.1;CTD-2005H7.2;RP11-762L8.6;PRSS23	0;0;0;0;28807;41947
ORF1_3_31344666_31349591_31366145_31370040_FF	0.0000382	0.001083302	-1.469998425	AC115283.1;THRAP3P1	75261;82793
ORF1_8_127857828_127859041_127947370_127950770_RF	0.00000143	0.000185505	-1.46679839	PVT1;PVT1_3;TMEM75;MIR1204;MIR1205	0;0;0;61801;9864
ORF1_8_128187181_128189922_128240174_128246477_FF	1.89E-09	9.194E-06	-1.466563373	RN7SKP226;MIR1208;LINC00824	0;36994;158793

ORF1_13_73346451_73348615_73424739_73428101_FF	1.61E-08	2.48902E-05	-1.462481319	RP11-309J13.1;MARK2P12;LINC00393;RNY1P8;LINC00392	0;0;0;119192;136144
ORF1_3_31220471_31226252_31459455_31461785_FR	4.72E-10	5.36929E-06	-1.46228798	RNA5SP127;AC115283.1;THRAP3P1;CNN2P6;STT3B	0;0;0;37822;70854
ORF1_9_21586051_21593014_21689015_21693759_FR	2.61E-08	2.86008E-05	-1.461008736	RP11-344A7.1;MIR31HG;KHSRPP1	0;26383;1418
ORF1_2_150139751_150145303_150209564_150211924_RF	0.000000531	0.000119506	-1.454231435	AC016682.1;AC007364.1;AC113610.1	0;280561;22969
ORF1_12_88924814_88929421_89019529_89023829_FF	3.52E-08	3.05706E-05	-1.451703554	RP11-13A1.1;RP11-900F13.2;RP11-13A1.2	0;322620;3929
ORF1_2_62561414_62564825_62729848_62731744_FF	0.00000088	0.000144644	-1.451406482	RSL24D1P2;AC092155.4;EHBP1;Y_RNA;PSAT1P2;AC007098.1	0;0;0;0;7981;94321
ORF1_13_73301186_73308791_73411813_73415867_FR	2.39E-10	4.33234E-06	-1.4443466	RP11-309J13.1;MARK2P12;LINC00393;RNY1P8;LINC00392	0;0;0;73927;148378
ORF1_22_19078251_19082547_19120233_19121325_RF	0.00000272	0.000261553	-1.443147178	DGCR2;DGCR12;AC004471.9;AC004471.10;TSSK1A	0;16409;205;2985;3555
ORF1_9_93218632_93223726_93274460_93278066_RF	0.000000112	5.52916E-05	-1.438635096	WNK2;RP11-370F5.4;C9orf129	0;70077;40134
ORF1_1_201477609_201480715_201530268_201534232_RF	0.00000666	0.000415567	-1.436310827	CSRP1;RP11-134G8.7;RP11-134G8.5;RP11-134G8.6;PHLDA3;NAV1	0;0;0;0;8373;88654
ORF1_9_21689015_21693759_21791167_21794897_FF	0.00000121	0.00017174	-1.432027005	KHSRPP1;RN7SL151P;RP11-344A7.1;MTAP	0;0;50340;7647
ORF1_7_156521340_156522478_156666661_156671039_FR	0.00000834	0.000464561	-1.431514183	LINC01006;LINC00244;AC005534.6;RNF32;AC005534.8;LMBR1;AC073133.1;RNU4-31P	0;0;0;0;0;0;75753;201040
ORF1_3_31220471_31226252_31236385_31244565_RF	5.49E-09	1.62439E-05	-1.42422338	RNA5SP127;CNN2P6;AC115283.1	0;37822;24443
ORF1_3_152927941_152931145_153141255_153143702_FF	0.000000438	0.000110496	-1.418511103	HMGN2P13;RP11-529G21.3;AC013251.1;RP11-529G21.2	0;0;8146;12810
ORF1_15_58229591_58234474_58325151_58334051_RF	0.000000384	0.000104511	-1.417859803	ALDH1A2;RP11-925D8.1;LIPC	0;28574;76519
ORF1_3_152927941_152931145_152997220_153000112_FR	0.000000534	0.000119533	-1.414311149	AC013251.1;HMGN2P13	8146;67167

ORF1_1_171800044_171801227_171918586_171920677_RR	1.39E-09	9.194E-06	-1.413693229	METTL13;RP4-612B18.3;RP4-612B18.1;DNM3;DNM3-IT1;SCARNA20;DNM3OS	0;0;0;0;0;31870;215855
ORF1_3_27085963_27092169_27173557_27176869_FF	1.86E-08	2.58705E-05	-1.408174302	NEK10;AC114877.1;MINOS1P3	0;331888;37948
ORF1_11_67397108_67399933_67578878_67580124_RF	4.08E-09	1.42592E-05	-1.402332303	RAD9A;PPP1CA;TBC1D10C;CARNS1;RPS6KB2;AP003419.16;PTPRCAP;CORO1B;GPR152;CABP4;TMEM134;AIP;MIR6752;PITPNM1;CDK2AP2;CABP2;RNU6-1238P;GSTP1	0;0;0;0;0;0;0;0;0;0;0;0;0;0;0;0;1798;3472
ORF1_16_19354331_19355598_19372415_19377934_RR	0.000166758	0.002649006	-1.401969768	CTA-363E6.2;CLEC19A;CTA-363E6.3	0;32187;14280
ORF1_1_206855273_206863926_207012040_207013449_RR	0.000000101	5.3475E-05	-1.401212709	IL20;IL24;FAIM3;RP11-564A8.8;RP11-564A8.4;PIGR;FCAMR;C4BPB;IL19;C1orf116	0;0;0;0;0;0;0;0;12293;5073
ORF1_2_234292543_234295680_234373128_234376985_FR	0.000000663	0.000131304	-1.398880341	AC097713.5;RP11-309M7.1;AC097713.3	0;68030;61344
ORF1_4_74622017_74627103_74792360_74795164_RR	0.00000115	0.000166373	-1.398083532	BTC;AC142293.3;HSPE1P23	0;32537;122659
ORF1_8_38795984_38800320_38924071_38927547_FF	0.00000141	0.000185032	-1.396954517	TACC1;RP11-723D22.2;RP11-723D22.3;PLEKHA2;Y_RNA;CTD-2544N14.3	0;0;0;0;51854;42814
ORF1_6_34241768_34243293_34274556_34276356_FF	0.0000301	0.000948451	-1.396865489	HMGA1;C6orf1;RP11-513I15.6;RPL35P2;MIR6835;NUDT3	0;0;0;0;1033;3324
ORF1_3_193440995_193448277_193553500_193557343_RR	0.000704675	0.006584756	-1.390261779	ATP13A4;ATP13A4-AS1;ATP13A5;RP11-175P19.2	0;0;62153;21470
ORF1_5_61009121_61015983_61116919_61125541_FR	0.000000233	8.12737E-05	-1.389502347	NDUFAF2;CTC-436P18.4;ERCC8;CTC-436P18.5	0;0;64049;20306
ORF1_16_18412767_18414502_18495366_18497101_FF	0.000000233	8.12737E-05	-1.383857042	NOMO2;MIR3670-2;MIR3179-1;MIR3179-3;RP11-457I16.4;MIR3179-3	0;0;0;791;36318;917
ORF1_1_156061088_156063372_156106979_156110773_RR	0.00000129	0.0001776	-1.379118037	RAB25;MEX3A;AL355388.1;LMNA;LAMTOR2;SEMA4A	0;0;0;0;2579;36594
ORF1_6_14589999_14592978_14738490_14739755_RF	0.0000614	0.001442275	-1.378784639	RP11-330A16.1;RNU6-793P;RP11-146I2.1;RP3-448I9.1;RP1-190J20.2	0;0;0;185711;363192
ORF1_1_183053245_183055209_183167430_183172905_RF	3.99E-10	5.36929E-06	-1.377309935	LAMC1;RP11-181K3.4;RNU6-41P;LAMC2	0;0;70928;13334

ORF1_13_29441818_29446142_29536770_29540750_RR	3.55E-08	3.06144E-05	-1.376678667	MTUS2;MTUS2-AS1;SLC7A1;RP11-351N4.4;UBL3	0;0;0;101950;223622
ORF1_11_86466088_86493462_86740416_86747101_FR	0.000074	0.001626009	-1.376471253	RP11-317J19.1;ME3;CTD-2005H7.1;CTD-2005H7.2;RP11-762L8.6;PRSS23	0;0;0;0;28807;43959
ORF1_20_46816898_46821498_46896496_46899476_FR	0.0000124	0.000580868	-1.37625961	RN7SKP33;RP11-323C15.1;EYA2;SLC2A10;RP11-323C15.2	0;0;0;80552;1668
ORF1_12_52177892_52180734_52307864_52312912_RR	0.000000872	0.000144482	-1.374679734	KRT80;C12orf80;LINC00592;METTL7AP1;KRT7;AC021066.1;RP3-416H24.1;KRT86;KRT87P;RP11-845M18.7;KRT81;RP11-845M18.6;AC078864.2;KRT83	0;0;0;0;0;0;0;0;0;0;0;63881;1390
ORF1_9_37919925_37923489_38002100_38004773_FR	0.000000249	8.26688E-05	-1.373971322	RP11-613M10.9;SHB;RNU7-124P;SLC25A51;AL161448.1	0;0;0;15573;141722
ORF1_11_86466088_86493462_86735082_86738567_FF	0.000191531	0.002885363	-1.373204313	RP11-317J19.1;ME3;CTD-2005H7.1;CTD-2005H7.2;RP11-762L8.6;PRSS23	0;0;0;0;28807;52493
ORF1_3_14207176_14212219_14431769_14438916_RF	0.000000256	8.33551E-05	-1.372697369	RP11-536I6.2;LINC01267;RNA5SP124;SLC6A6;LSM3;GRIP2	0;0;0;0;6058;50196
ORF1_18_75089410_75092526_75218902_75221270_RR	0.00000319	0.000284714	-1.371787897	RP11-405M12.2;ZADH2;TSHZ1;RP11-405M12.4;RP11-321M21.4	0;0;0;15206;165787
ORF1_3_30633318_30636860_30722395_30727686_RR	0.000000751	0.000138916	-1.370901203	TGFBR2;RP11-1024P17.1;GADL1;AC096921.2;MIR466	0;0;0;6522;434019
ORF1_12_53992251_53994278_54020039_54026366_FR	0.00000511	0.000364985	-1.370448259	RP11-834C11.12;HOXC6;HOXC-AS2;HOXC9;HOXC-AS1;HOXC8;HOXC4;RP11-834C11.14;AC012531.25;MIR196A2;HOXC5;HOXC10	0;0;0;0;0;0;0;0;400;6488;1973
ORF1_3_31220471_31226252_31407326_31412414_FR	0.000000673	0.000132408	-1.369430013	RNA5SP127;AC115283.1;CNN2P6;THRAP3P1	0;0;37822;40419
ORF1_8_93886900_93893089_93985677_93987921_FF	0.0000019	0.000214555	-1.369140328	PDP1;RP3-388N13.5;MIR378D2;PSMA2P2;RPL34P18;AC105081.1;CTC-788C1.2;RP3-388N13.2;KB-1184D12.1	0;0;0;0;0;0;0;36826;109844
ORF1_11_86466088_86493462_86514624_86517539_FR	0.000622564	0.006083755	-1.368004317	RP11-317J19.1;ME3;RP11-762L8.6;CTD-2005H7.1	0;0;28807;185561
ORF1_12_116550240_116554459_116642258_116646284_FR	0.00000136	0.000183173	-1.365251961	MAP1LC3B2;RP11-497G19.7;RP11-497G19.3;LINC00173;RP11-497G19.1	0;0;0;13723;15299

ORF1_2_234128031_234133295_234373128_234376985_RR	0.000000441	0.000110627	-1.365062257	RP11-309M7.1;AC097713.5;AC006037.2;AC097713.3	0;0;18551;61344
ORF1_8_127286961_127289125_127517840_127519038_FF	0.00000198	0.000219	1.418217837	CASC8;RP11-382A18.3;CASC21;CCAT2;POU5F1B;CCAT1;AC108925.1	0;0;0;0;0;67874;111246
ORF1_8_3758367_3759691_3786645_3789952_RR	0.0000558	0.001358	1.418765023	CSMD1;RNA5SP251;PAICSP4	0;57740;997381
ORF1_12_13456395_13466337_13573247_13579043_FF	0.000000038	0.000032	1.419197868	RNU6-590P;RP11-4N23.1;GRIN2B;RP11-4N23.4;RNA5SP353;AC007535.1	0;0;0;0;15395;8838
ORF1_10_112427560_112432424_112517647_112519444_FF	0.000000102	0.0000535	1.421664591	ACSL5;ZDHHC6;VTI1A;RP11-324O2.3;RP11-214N15.5	0;0;0;1972;29189
ORF1_15_85690034_85695202_85840592_85842210_RR	0.00000372	0.000307	1.422921775	AKAP13;RP11-815J21.1;RP11-158M2.2;RP11-158M2.3;RP11-158M2.4;RP11-158M2.5;KLHL25;MIR1276;RP11-158M2.6;RNU6-231P;MIR548AP;RP11-815J21.4;RP11-23A22.1	0;0;0;0;0;0;0;0;0;0;19087;116508
ORF1_10_109690786_109701852_109729407_109735286_RF	2.44E-08	0.0000286	1.42466586	RP11-566F5.1;RP11-451M19.2	9746;72366
ORF1_13_73435053_73437099_73484222_73486544_RF	4.43E-08	0.0000346	1.424866474	LINC00393;MARK2P12;LINC00392	0;26702;77701
ORF1_3_31220471_31226252_31279538_31283760_FF	0.0000519	0.001302	1.424950179	RNA5SP127;AC115283.1;CNN2P6;THRAP3P1	0;0;37822;169073
ORF1_20_2248290_2250880_2303567_2312197_FR	2.9E-09	0.0000116	1.425670761	TGM3;RP11-128M1.1;TGM6	0;35140;68712
ORF1_20_8765158_8766621_8858679_8866038_FF	0.00000202	0.000222	1.432019215	PLCB1;RNU105B;AL031655.1;RP5-859I17.2	0;0;154919;63858
ORF1_9_129574873_129578569_129635562_129636688_FF	5.17E-10	0.00000542	1.433867974	RP11-492E3.2;NTMT1;C9orf50;ASB6;RP11-492E3.51;RP11-483H20.4	0;0;0;0;61188;3789
ORF1_6_39460001_39466858_39554127_39556478_RF	0.00000332	0.000291	1.434985984	KIF6;E2F4P1;RP1-137F1.3;RNU1-54P	0;0;105830;63868
ORF1_13_73327022_73333613_73435053_73437099_FR	0.0000234	0.000813	1.435433252	RP11-309J13.1;MARK2P12;LINC00393;RNY1P8;LINC00392	0;0;0;99763;127146
ORF1_6_39460001_39466858_39589439_39594681_RF	0.0000128	0.00059	1.435464944	KIF6;E2F4P1;RP1-137F1.3;RNU1-54P	0;0;105830;25665

ORF1_1_94243796_94250808_94301164_94309744_FR	0.000000315	0.0000937	1.437106397	ARHGAP29;RP11-148B18.1;GAPDHP29;RN7SL440P;RP11-148B18.4	0;0;0;92717;8736
ORF1_6_52301314_52302830_52356722_52357908_FF	0.0000404	0.001125	1.438151583	PAQR8;MCM3	3514;16434
ORF1_4_152108183_152110614_152172738_152175161_RR	0.000000034	0.0000304	1.438566626	RP11-18H21.2;RP11-18H21.1;RP11-18H21.3	0;3464;32261
ORF1_4_55405022_55407291_55545903_55548097_RR	0.001123461	0.008883	1.440542348	TMEM165;Y_RNA;CLOCK;Y_RNA;RN7SKP30;RP11-528I4.2;SRD5A3-AS1;PDCL2	0;0;0;0;0;0;9176;8429
ORF1_9_69174078_69179874_69282353_69289706_RF	0.0000166	0.000671	1.44103967	TJP2;RP11-265B8.5;BANCR	0;65862;6977
ORF1_12_93013996_93019448_93102345_93106201_FR	2.02E-08	0.0000264	1.445482624	RP11-511B23.1;RP11-511B23.2;Y_RNA;RP11-511B23.4;RPL41P5;RP11-202G11.2;AC138123.1;RNU6-1329P;NACAP3	0;0;0;0;0;0;0;61252;17863
ORF1_8_127080396_127086850_127286961_127289125_RF	0.0000604	0.001429	1.448294324	PCAT2;RP11-255B23.1;CASC19;CCAT1;PCAT1;CASC8;RP11-382A18.3	0;0;0;0;59383;693;3109
ORF1_12_20768546_20775250_20818554_20821322_FF	0.000000531	0.00012	1.448711744	SLCO1B3;RP11-545J16.1;SLCO1C1;RP11-80N2.2	0;0;15161;23744
ORF1_8_100963172_100967658_101138999_101140681_FR	0.0000569	0.001376	1.44915584	FLJ42969;KB-1460A1.1;KB-1460A1.2;RN7SKP249;KB-1460A1.3;RN7SL685P;KB-1460A1.5	0;0;0;0;0;4993;26125
ORF1_8_29599983_29603803_29671847_29673621_RF	0.000000254	0.000253	1.453250539	AC069181.1;RPL17P33;RP11-486M23.3;LINC00589	0;0;11558;302
ORF1_13_73289113_73297526_73435053_73437099_RR	0.00003	0.000947	1.453260019	RP11-309J13.1;MARK2P12;LINC00393;RNY1P8;LINC00392	0;0;0;61854;127146
ORF1_5_74512329_74516271_74623804_74625089_FF	2.49E-08	0.0000286	1.453859129	LINC01331;CTD-2275D24.1;ENC1	0;172178;2318
ORF1_19_46026038_46028027_46038294_46045979_RR	0.00000151	0.000191	1.454877584	CTC-344H19.4;IGFL4;PGLYRP1;AC007193.6;CCDC61	0;0;2974;55144;4721
ORF1_4_152108183_152110614_152189282_152191052_RR	4.31E-08	0.000034	1.455911913	RP11-18H21.2;RP11-18H21.1;RP11-18H21.3	0;3464;16370
ORF1_6_12246667_12248138_12375235_12377947_FR	0.000000624	0.000129	1.460208798	EDN1;SUMO2P12;HIVEP1;RN7SKP293	0;0;81669;28540

ORF1_5_70995583_71002742_71084688_71085983_RR	0.000000835	0.000142	1.460285483	NAIP;GTF2H2;RP11-195E2.1;SMN1;RP11-1280N14.3	0;0;0;41642;16916
ORF1_3_37850166_37859561_37922029_37924342_RF	0.000000821	0.000142	1.461806616	ITGA9-AS1;CTDSPL;ITGA9;MIR26A1	0;0;26653;45063
ORF1_15_71449255_71457687_71567140_71571578_RR	0.00000685	0.000421	1.462480028	THSD4;RP11-1123I8.1;RP11-592N21.2;AC104938.1	0;0;100785;201033
ORF1_13_33598267_33602312_33679199_33682012_RR	0.000138903	0.002363	1.462774218	RP11-141M1.3;RP11-37L2.1;RP11-179A7.2;AL139383.1;RFC3	0;0;0;219702;136038
ORF1_6_39460001_39466858_39607275_39611834_RF	0.000000625	0.000129	1.465846298	KIF6;E2F4P1;RP1-137F1.3;RNU1-54P	0;0;105830;8512
ORF1_3_124580063_124582715_124604361_124610958_FR	0.000000286	0.0000887	1.472239151	KALRN;RNU6-143P;AC080008.1	0;172281;85981
ORF1_2_135944935_135947001_136069708_136077065_RR	6.01E-08	0.0000397	1.473418418	DARS;DARS-AS1;AC011999.1;AC068492.1	0;0;45958;828
ORF1_4_99346122_99351523_99416691_99418636_FR	0.000000304	0.0000922	1.477811471	ADH1C;ADH7;ADH1B;RP11-696N14.3	0;0;24681;50963
ORF1_11_16883235_16885791_16898189_16901066_RR	0.000300534	0.003813	1.478446515	PLEKHA7;RN7SKP90;RP11-466H18.1	0;40649;73628
ORF1_X_46329006_46332897_46492211_46496918_FR	0.0000127	0.000588	1.478913083	RP11-75A9.1;GAPDHP65;KRBOX4;RP11-75A9.3;LINC01186;ZNF674	0;0;0;0;1362;810
ORF1_12_92983471_92985747_93013996_93019448_RF	0.00000458	0.000342	1.481115859	RP11-511B23.1;RP11-511B23.2;RNU6-1329P;Y_RNA	0;0;30727;2159
ORF1_11_80010400_80012110_80052904_80065666_RF	0.0000204	0.000753	1.482737691	RP11-885L14.1;CTD-2555I5.1	20771;255955
ORF1_6_39460001_39466858_39541511_39551472_RF	0.00000156	0.000196	1.482827145	KIF6;RP1-137F1.3;E2F4P1	0;105830;2340
ORF1_20_2248290_2250880_2299895_2302840_FF	2.93E-08	0.0000286	1.485976898	TGM3;RP11-128M1.1;TGM6	0;35140;78069
ORF1_13_73411813_73415867_73484222_73486544_RF	2.38E-08	0.0000286	1.497424179	LINC00393;MARK2P12;LINC00392	0;3462;77701
ORF1_20_4055043_4057398_4141331_4145409_FR	0.000000198	0.0000737	1.501067612	RPL21P2;RP11-352D3.2;SMOX;FTLP3;LINC01433	0;0;0;30600;47657

ORF1_10_20998825_21002083_21096009_21097256_FR	1.01E-10	0.00000344	1.504902192	NEBL;EIF4BP2;RP11-56H7.2;NPM1P30	0;0;157266;13427
ORF1_17_55861684_55873686_55959366_55961176_FR	0.0000357	0.001044	1.507109304	RP11-763E3.1;PCTP;AC011073.1	0;18855;93612
ORF1_8_29599983_29603803_29661949_29668203_RF	0.0000018	0.000209	1.509321731	AC069181.1;RPL17P33;RP11-486M23.3;LINC00589	0;0;11558;5720
ORF1_13_106465011_106468917_106520293_106525786_RF	0.0000722	0.001602	1.510083438	EFNB2;RP11-272L14.2;RPL35P9;ARGLU1	0;0;38890;15888
ORF1_18_63960301_63966443_64058257_64068334_FF	0.000763573	0.006934	1.512138366	HMSD;SERPINB8;SERPINB10;RPL12P39	0;0;24191;3825
ORF1_5_70995583_71002742_71085983_71088174_RR	0.0000312	0.000967	1.52252304	NAIP;GTF2H2;RP11-195E2.1;SMN1;RP11-1280N14.3	0;0;0;41642;14725
ORF1_13_46623986_46627474_46717434_46721309_FF	0.00000589	0.000391	1.523615557	LRCH1;RP11-165D6.1;FKBP1AP3;ESD	0;0;118551;49948
ORF1_1_8019357_8024615_8152445_8155172_FF	0.00000519	0.000368	1.52589905	ERRFI1;RP11-431K24.1;CTA-215D11.4;PARK7;RP11-431K24.2	0;0;0;33853;34653
ORF1_14_70711283_70715221_70826394_70830033_FF	0.000000174	0.0000696	1.5274744	LINC01269;MAP3K9;AC004816.1;RP6-65G23.3;RP6-65G23.5;TTC9;RP1-292L20.2	0;0;0;0;0;35924;28812
ORF1_6_35273392_35274796_35355832_35362162_FF	0.00005	0.001276	1.531862706	ZNF76;RP3-329A5.1;DEF6;PPARD;SCUBE3;MKRNP2	0;0;0;0;20314;80883
ORF1_2_234370725_234373128_234383545_234388702_FF	0.0000017	0.000678	1.53296092	AC097713.5;AC097713.3	40888;49627
ORF1_10_96209904_96214032_96274838_96286475_FF	0.000000016	0.0000666	1.535452306	BLNK;ZNF518A;RP11-35J23.1	0;4617;6211
ORF1_1_94060570_94064104_94081020_94084795_RF	0.0000011	0.000163	1.535480941	ABCA4;RP5-837O21.2;RP11-78O9.1	0;125327;60317
ORF1_5_10308625_10310916_10408164_10410166_FR	0.000000815	0.000142	1.536937449	Y_RNA;CTD-2256P15.3;CTD-2256P15.2;38777;AC012640.1;CMBL;ROPN1L-AS1	0;0;0;0;0;600;31125
ORF1_8_26155314_26158948_26212756_26223581_RF	0.00000112	0.000164	1.549068684	RNA5SP258;RP11-98P2.1	18317;31303
ORF1_8_93886900_93893089_94052891_94056029_FR	0.000000853	0.000144	1.555229232	PDP1;RP3-388N13.5;MIR378D2;PSMA2P2;RPL34P18;AC105081.1;CTC-788C1.2;RP3-388N13.2;KB-1184D12.1	0;0;0;0;0;0;0;36826;41736

ORF1_8_125042388_125043839_125227687_125229276_FR	0.000000607	0.000127	1.557775659	KIAA0196;KIAA0196-AS1;NSMCE2;SQLE;RN7SL329P	0;0;0;20106;41329
ORF1_7_17237706_17242932_17510943_17517538_FF	0.000533932	0.005492	1.560530422	AC003075.4;AHR;SNORA63;AC019117.1;AC019117.2;AC017060.1;Metazoa_SRP;AC006482.1	0;0;0;0;0;0;31779;162012
ORF1_13_44297328_44312567_44329948_44335173_RR	0.0000169	0.000677	1.569526706	RP11-269C23.5;RP11-478K15.6	34193;54137
ORF1_8_93427748_93432802_93580613_93588814_FR	0.000403405	0.004592	1.569728786	LINC00535;RNA5SP274;RP11-388K12.2;ZNF317P1	0;0;129773;57253
ORF1_3_4954443_4957431_4990259_4996900_FF	0.00023583	0.003276	1.585851437	BHLHE40-AS1;BHLHE40;AC018816.3;RNF10P1	0;0;67151;71777
ORF1_9_85940401_85942280_86061463_86069522_RR	0.000882078	0.007624	1.587068384	NAA35;GOLM1;RP11-213G2.5;RP11-379P1.4	0;0;3803;63101
ORF1_19_46038294_46045979_46117700_46123708_RF	0.000000398	0.000106	1.596616101	IGFL4;AC007193.6;CTC-344H19.6;IGFL3;CTC-344H19.4;TGIF1P1	0;0;0;0;8134;13039
ORF1_6_137117975_137119403_137232223_137235679_RR	0.000000092	0.0000507	1.597043435	IL22RA2;IFNGR1;IL20RA;OLIG3	0;0;72796;256524
ORF1_6_151029858_151031265_151049153_151055997_RF	0.000598484	0.005904	1.599175338	MTHFD1L;AL133260.1;RP1-292B18.3;RNU6-302P;RP1-292B18.4	0;0;0;90531;32107
ORF1_1_94067082_94069443_94081020_94084795_RF	5.87E-08	0.0000393	1.601711361	ABCA4;RP5-837O21.2;RP11-78O9.1	0;131839;60317
ORF1_18_63960301_63966443_64015608_64016634_FR	0.000584133	0.005816	1.603258433	HMSD;SERPINB8;SERPINB10;RPL12P39	0;0;24191;55525
ORF1_4_38166364_38167703_38275089_38278473_RF	4.35E-11	0.00000307	1.604163394	AC098680.1;TBC1D1;RP11-600L4.1	0;27190;8522
ORF1_2_45917848_45921108_45965825_45971368_FF	0.0000138	0.000613	1.618321889	PRKCE;U51244.2;AC017078.1	0;242373;32575
ORF1_2_227807724_227811007_227923203_227932124_RF	0.00000166	0.0002	1.620528196	CCL20;TDGF1P2;DAW1;SNORA25;AC073065.3;SPHKAP	0;0;0;0;3020;47827
ORF1_5_61116919_61125541_61216012_61225000_RF	0.0000904	0.001821	1.628478289	NDUFAF2;CTC-436P18.5;SMIM15;CTC-436P18.1;CTC-436P18.3;CTC-436P18.4;ZSWIM6	0;0;0;0;0;167;107274
ORF1_8_142955054_142956798_143012831_143021167_FF	0.000000751	0.000139	1.64264573	RP11-273G15.2;CDC42P3;LY6E;CYP11B2;C8orf31	0;0;0;37212;18043

ORF1_2_188185903_188189441_188290117_188294825_RR	1.54E-08	0.0000247	1.643027525	LINC01090;AC104131.1;RNA5SP114;GULP1;ST13P2;MIR561	0;0;0;0;359455;2668
ORF1_15_67079527_67081854_67195948_67198335_RF	0.00000018	0.0000703	1.652685053	SMAD3;RP11-342M21.2;RP11-798K3.2;AAGAB	0;0;20275;2699
ORF1_2_43291539_43295772_43398047_43401051_FF	0.0000126	0.000587	1.658290904	THADA;AC010883.5;RNU6-958P	0;58146;7257
ORF1_15_67146248_67149065_67195948_67198335_RF	5.18E-08	0.0000371	1.661979894	SMAD3;RP11-342M21.2;RP11-798K3.2;AAGAB	0;0;86996;2699
ORF1_7_116779665_116782230_116835951_116839465_FF	0.000000431	0.00011	1.66722655	MET;CAPZA2;LINC01510;AC002543.2	0;0;164846;33990
ORF1_8_93985677_93987921_94110777_94115884_FF	0.000000725	0.000138	1.692946545	KB-1184D12.1;CTC-788C1.2;CDH17	0;10902;11288
ORF1_8_125227687_125229276_125289724_125291437_RR	0.00000165	0.0002	1.711754195	NSMCE2;RN7SL329P;KIAA0196;RP11-550A5.2	0;0;135848;56760
ORF1_17_57291624_57293132_57370262_57372885_FR	0.00000533	0.000374	1.727792433	MSI2;RP11-426D19.1;RP11-226M10.3	0;11608;75334
ORF1_4_186767785_186774939_186803232_186810282_RF	0.00000528	0.000371	1.758078328	FAT1;RP11-11N5.1	41064;80688
ORF1_12_75758917_75764956_76021287_76023803_RR	0.00000295	0.000273	1.764356689	RP11-114H23.1;RP11-290L1.6;RN7SL734P;RP11-290L1.5;RP11-290L1.7;AC131012.1;PHLDA1	0;0;0;0;0;34377;1645
ORF1_15_67123957_67126058_67195948_67198335_RF	5.65E-08	0.0000387	1.771428401	SMAD3;RP11-342M21.2;RP11-798K3.2;AAGAB	0;0;64705;2699
ORF1_1_53923440_53929801_53955849_53957797_RF	0.000144017	0.002419	1.772793664	HSPB11;LRRC42;DIO1;HNRNPA3P12	0;0;12355;17173
ORF1_4_38173096_38176661_38275089_38278473_RF	0.000000134	0.0000615	1.772982825	AC098680.1;TBC1D1;RP11-600L4.1	0;33922;8522
ORF1_10_15474797_15476116_15598170_15604222_FR	0.0000197	0.000737	1.786426594	ITGA8;FAM171A1;FAM188A	0;103736;173949
ORF1_12_95030095_95034137_95200229_95202613_FR	0.000000064	0.0000413	1.789451355	NR2C1;FGD6;RP11-129B9.2;RP11-129B9.1;NDUFA12;VEZT	0;0;0;0;26326;15134
ORF1_9_92275780_92277607_92292748_92297879_RF	0.0000228	0.000801	1.812045837	IARS;MIR3651;SNORA84;NOL8;FKSG56;CENPP	0;0;0;0;48406;27606

ORF1_9_107207372_107209526_107328595_107331976_FF	0.00000015	0.0000642	1.81379858	RAD23B;AL137852.1;RP11-417L14.1;LINC01509	0;0;36225;88309
ORF1_17_72593871_72597525_72705796_72713111_FR	0.0000209	0.000763	1.819724722	LINC00511;RPL32P33;CTD-3010D24.3;SLC39A11;RN7SKP180;RP11-57A1.1;AC080037.1	0;0;0;0;0;165020;1273
ORF1_19_15554708_15555923_15715613_15718003_RR	0.000000475	0.000114	1.820469066	CYP4F23P;AD000091.3;RPL23AP2;AD000091.2;CYP4F8;CYP4F3;CYP4F10P;CYP4F12;CYP4F22;OR10H2	0;0;0;0;0;0;0;0;2392;10018
ORF1_20_49630304_49633529_49693997_49696402_FR	0.0000011	0.000163	1.822125395	B4GALT5;AL118525.1;Metazoa_SRP	0;43531;25548
ORF1_3_31236385_31244565_31366145_31370040_FF	0.00000037	0.000103	1.851232006	AC115283.1;RNA5SP127;THRAP3P1	0;7539;82793
ORF1_14_54172832_54178414_54204938_54208737_RR	0.000000988	0.000155	1.852947194	ATP5C1P1;CDKN3	180838;188113
ORF1_8_127283149_127286736_127379773_127382742_RF	0.000000761	0.000139	1.863344491	CASC8;RP11-382A18.3;CASC21;CCAT1;CCAT2	0;0;0;64062;17658
ORF1_13_44177870_44185274_44297328_44312567_FR	0.00000732	0.000434	1.884141848	SMIM2-AS1;RP11-478K15.7;MIR8079;RP11-478K15.6;SMIM2;RP11-269C23.5	0;0;0;0;16614;56799
ORF1_12_88808618_88814696_88924814_88929421_RR	0.0000035	0.000299	1.888053598	RP11-13A1.1;RP11-900F13.2	81261;206424
ORF1_19_15715613_15718003_15941737_15958190_RR	0.00020337	0.002989	1.93428938	OR10H2;OR10H3;CYP4F24P;LLNLR-249E10.1;OR10H5;OR10H1;ZNF861P;AC004510.3;UCA1;UCA1;AC004791.2;CYP4F36P;CYP4F2;AC005336.5;LLNLF-158E9.1;AC005336.4;CYP4F11;OR10H4;CYP4F12;CYP4F9P	0;0;0;0;0;0;0;0;0;0;0;0;0;0;0;0;18440;21646
ORF1_11_86595548_86597707_86864298_86870664_FF	0.00000663	0.000414	1.946964291	RP11-317J19.1;ME3;CTD-2005H7.1;CTD-2005H7.2;PRSS23;AP000654.5;OR7E13P;AP000654.4;OR7E2P;RP11-762L8.6;RP11-736K20.5	0;0;0;0;0;0;0;0;158267;21551
ORF1_20_53779660_53781229_53887494_53889551_RF	0.00000163	0.000199	1.972009066	AC006076.1;SUMO1P1;RNU7-14P;BCAS1	0;0;110903;47227
ORF1_X_13222287_13224956_13303825_13307162_RF	0.00000003	0.0000288	1.97632727	GS1-600G8.5;RP11-791M20.1;GS1-600G8.3	0;127715;3491
ORF1_12_75781130_75790040_76067494_76074003_FF	0.00000338	0.000293	1.987338943	RP11-114H23.1;RP11-290L1.6;RN7SL734P;RP11-290L1.5;RP11-290L1.7;PHLDA1;RP11-	0;0;0;0;0;0;0;0;56590;41709

				290L1.3;RP11-290L1.2;NAP1L1;RP11-290L1.4;AC131012.1;RP11-453D16.1	
ORF1_11_69123218_69124400_69217270_69219761_FF	0.00000925	0.00049	2.039392053	RP11-554A11.8;RP11-554A11.9;RP11-554A11.7;MYEOV	0;0;14125;74378
ORF1_13_73301186_73308791_73411813_73415867_FF	0.00000243	0.000247	2.052691746	RP11-309J13.1;MARK2P12;LINC00393;RNY1P8;LINC00392	0;0;0;73927;148378
ORF1_8_93868701_93870106_93985677_93987921_FR	0.00000163	0.000199	2.25143761	PDP1;RP3-388N13.5;MIR378D2;PSMA2P2;RPL34P18;AC105081.1;CTC-788C1.2;RP3-388N13.2;KB-1184D12.1	0;0;0;0;0;0;0;18627;109844
ORF1_9_21689015_21693759_21770077_21774135_RF	0.042661557	1	-2.50241894	KHSRPP1;RN7SL151P;RP11-344A7.1;MTAP	0;0;50340;28409
ORF1_2_226385191_226398167_226558209_226561297_FF	0.026534485	1	-1.583810543	MIR5702;AC068138.1	97414;199821
ORF1_16_15450937_15453244_15568679_15570721_FR	0.006656119	1	-1.484156163	RP11-1021N1.1;C16orf45;MPV17L;KIAA0430	0;0;37670;23666
ORF1_6_113321989_113323652_113358439_113364850_RR	0.009163543	1	-1.482884568	RP11-437J19.1;SOCS5P5;RP1-124C6.1	0;97918;63691
ORF1_1_143747166_143748632_143780252_143783873_FF	0.031959768	1	-1.46662555	RP11-403I13.4;FAM91A3P;RP11-403I13.5;RP11-403I13.10	0;0;7661;504
ORF1_4_14863870_14870511_15000881_15003251_FR	0.025485477	1	-1.462722543	LINC00504;CPEB2-AS1;CPEB2;SNORA63;RP11-665G4.1	0;0;0;173039;1692
ORF1_8_93868701_93870106_93985677_93987921_FF	0.008151569	1	-1.457135515	PDP1;RP3-388N13.5;MIR378D2;PSMA2P2;RPL34P18;AC105081.1;CTC-788C1.2;RP3-388N13.2;KB-1184D12.1	0;0;0;0;0;0;0;18627;109844
ORF1_2_113209902_113215780_113275966_113277494_FR	0.017856643	1	-1.456428166	PAX8-AS1;PAX8;RP11-65I12.1;PSD4;IGKV1OR2-108	0;0;0;507;128903
ORF1_17_18606283_18608952_18726686_18728395_FF	0.034475789	1	-1.449511943	CCDC144B;TBC1D28;RP11-815I9.3;AC026271.5;ZNF286B;RP11-815I9.4;FOXO3B;UBE2SP2;TRIM16L;RP11-815I9.5;Metazoa_SRP;FBXW10	0;0;0;0;0;0;0;0;0;928;15630
ORF1_8_93747157_93749988_93868701_93870106_RF	0.005813127	1	-1.438736473	TMEM67;RP3-388N13.3;RP3-388N13.2;PDP1;RP11-10N23.5;RP3-388N13.5	0;0;0;0;2624;45629
ORF1_2_207160403_207162770_207312461_207318286_RR	0.005449014	1	-1.437770549	KLF7;MIR7845;AC007879.1;AC007879.2;AC007879.4;AC007879.3;AC007879.7;AC007879.5;AC007879.6;MIR1302-4;KLF7-IT1;CREB1	0;0;0;0;0;0;0;0;0;38360;211452

ORF1_1_143747166_143748632_143780252_143783873_FR	0.005968065	1	-1.434144495	RP11-403I13.4;FAM91A3P;RP11-403I13.5;RP11-403I13.10	0;0;7661;504
ORF1_1_116503077_116513406_116627241_116630872_RF	0.025359513	1	-1.432858812	CD58;NAP1L4P1;MIR548AC;IGSF3;AL355794.1;RP5-1086K13.1;MIR320B1	0;0;0;0;0;3866;40878
ORF1_2_207160403_207162770_207265456_207267943_RR	0.008976136	1	-1.414835614	KLF7;MIR7845;AC007879.1;AC007879.2;AC007879.4;AC007879.3;AC007879.7;AC007879.5;AC007879.6;KLF7-IT1;MIR1302-4	0;0;0;0;0;0;0;0;0;38360;1333
ORF1_22_43659592_43667556_43746279_43747474_FR	0.007094146	1	-1.401032887	EFCAB6;EFCAB6-AS1;RP3-388M5.8	0;122476;11726
ORF1_1_143713002_143715279_143780252_143783873_RR	0.015381718	1	-1.397718048	RNU1-92P;AC239800.1;RNVU1-18;RP11-403I13.5;RP11-403I13.4;FAM91A3P;RNVU1-17;RP11-403I13.10	0;0;0;0;0;0;13384;504
ORF1_12_11012518_11022643_11033600_11037094_RR	0.004788341	1	-1.390276589	PRH1;PRH1-PRR4;PRR4;TAS2R14;TAS2R19;TAS2R31;TAS2R20;TAS2R63P	0;0;0;0;0;0;14644;11239
ORF1_22_43659592_43667556_43716745_43722322_FR	0.016303296	1	-1.380307217	EFCAB6;EFCAB6-AS1;RP3-388M5.8	0;122476;36878
ORF1_1_143731633_143735936_143780252_143783873_RR	0.031140632	1	-1.362196505	RP11-403I13.5;RP11-403I13.4;FAM91A3P;RNVU1-18;RP11-403I13.10;AC239800.1	0;0;0;2064;504;2366
ORF1_6_42077189_42078919_42218973_42222973_RF	0.003854841	1	-1.35985235	TAF8;RP11-7K24.3;C6orf132;GUCA1A;RP1-139D8.6;GUCA1B;MRPS10;CCND3;TRERF1	0;0;0;0;0;0;0;26833;1959
ORF1_8_8307248_8309141_8529093_8530943_RF	0.016157175	1	-1.342913514	SGK223;CTA-398F10.1;CTA-398F10.2;FAM86B3P;CTD-3023L14.3	0;0;0;62384;24796
ORF1_22_43659592_43667556_43685039_43686199_FR	0.016034408	1	-1.342268133	EFCAB6;EFCAB6-AS1;RP3-388M5.8	0;122476;73001
ORF1_1_116484855_116489949_116627241_116630872_RF	0.0129173	1	-1.33353649	RP5-1086K13.1;CD58;NAP1L4P1;MIR548AC;IGSF3;AL355794.1;RP4-655J12.4;MIR320B1	0;0;0;0;0;0;6014;40878
ORF1_2_28600362_28606396_28739083_28741489_RF	0.04968129	1	-1.326691414	PLB1;AC074011.2;RNA5SP89;AC097724.3;SNRPGP7;PPP1CB	0;0;0;0;140002;10152
ORF1_6_12190780_12196786_12375235_12377947_RR	0.010270237	1	-1.32282566	EDN1;SUMO2P12;HIVEP1;RN7SKP293	0;0;25782;28540
ORF1_11_12000738_12002447_12153671_12160688_FF	0.035394393	1	-1.311668515	DKK3;RP13-631K18.2;RP13-631K18.3;AC124276.1;RP13-631K18.5;MICAL2;USP47;MIR6124	0;0;0;0;0;0;41416;2996

ORF1_1_112908654_112912491_113056685_113057969_FR	0.014903415	1	-1.308666044	SLC16A1;AKR7A2P1;SLC16A1-AS1;RP11-389O22.1;RP11-31F15.2;RP3-522D1.2;LRIG2	0;0;0;0;0;17733;15241
ORF1_2_28576879_28580094_28739083_28741489_RF	0.041971309	1	-1.3077024	PLB1;AC074011.2;RNA5SP89;AC097724.3;SNRPGP7;PPP1CB	0;0;0;0;116519;10152
ORF1_1_94643992_94645758_94797386_94803287_RR	0.031655923	1	-1.306144095	LINC01057;KATNBL1P2;PGBD4P7;AC093117.1;MIR378G;RP11-86H7.7;SLC44A3	0;0;0;0;0;19809;17056
ORF1_5_74127901_74129338_74325511_74329920_RF	0.034432297	1	-1.303045717	LINC01335;RN7SL814P;CTD-2275D24.1;LINC01333;LINC01332;CTD-2292M14.1;LINC01331	0;0;0;0;0;24686;39457
ORF1_10_33504425_33507653_33649397_33655460_RF	0.000258492	1	-1.2999638	RP11-476F14.1;RP11-90B22.1;RP13-259N13.2	0;162521;29296
ORF1_10_46427799_46431159_46513779_46520085_FF	0.024555352	1	-1.298522249	ANXA8L1;NPY4R;HNRNPA1P33;GPRIN2	0;0;11387;28960
ORF1_1_116481182_116484855_116627241_116630872_RF	0.026301409	1	-1.295889575	RP5-1086K13.1;CD58;NAP1L4P1;MIR548AC;IGSF3;AL355794.1;RP4-655J12.4;MIR320B1	0;0;0;0;0;0;2341;40878
ORF1_10_22810599_22813175_22902674_22906733_FR	0.004116381	1	-1.287459379	ARMC3;RNU6-413P	21292;23101
ORF1_8_29661949_29668203_29765923_29776926_RF	0.018633083	1	-1.284791288	LINC00589;AC145110.1;RPL17P33;RP11-94H18.1	0;0;29306;38079
ORF1_8_3756152_3758367_3969249_3970970_FR	0.022124159	1	-1.281994331	CSMD1;RNA5SP251;PAICSP4	0;55525;816363
ORF1_8_102789661_102792716_102876735_102877950_RF	0.016872211	1	-1.274489783	KB-1732A1.1;AZIN1;KB-1254G8.1;KB-1507C5.2;AP003356.1;KB-1507C5.3	0;0;0;0;55943;13927
ORF1_17_80002331_80003967_80070704_80072351_RF	0.010226283	1	-1.27298952	TBC1D16;CTD-2529O21.2;CCDC40;CTD-2529O21.1;GAA	0;0;0;9491;29206
ORF1_13_109265513_109269608_109370464_109378162_RF	0.003872426	1	-1.272107901	RP11-54H7.4;LINC00370;LINC01067;MYO16;LINC00399	0;0;0;57507;22535
ORF1_4_74534540_74542386_74673362_74675454_FF	0.030135209	1	-1.267925129	AC142293.3;AREG;BTC	0;79532;69306
ORF1_7_55132651_55134082_55175644_55179436_RR	0.036206053	1	1.291851148	EGFR;RP4-791C19.1;EGFR-AS1	0;194468;315
ORF1_3_149353577_149357857_149407044_149409937_FR	0.029038099	1	1.329492313	RP11-2G17.1;TM4SF1;TM4SF1-AS1;RP11-278L15.2;TM4SF18;RP11-167H9.1	0;0;0;0;19164;23108

ORF1_1_183124731_183128683_183167430_183172905_RF	0.020860079	1	1.378744308	LAMC1;RP11-181K3.4;RNU6-41P;LAMC2	0;0;142414;13334
ORF1_7_155235541_155238765_155374563_155379862_RR	0.03068305	1	1.381534016	AC099552.2;AC144652.1;INSIG1;BLACE;RP11-1430O6.1;AC008060.7	0;0;0;0;11691;2215
ORF1_15_80632363_80638226_80777715_80785550_FR	0.036025502	1	1.382881382	RNU6-380P;ABHD17C;RP11-28H5.2;CEMIP;ARNT2;MIR549A	0;0;0;0;34427;56429
ORF1_15_60380602_60382233_60472233_60475235_FF	0.033803501	1	1.388139524	ANXA2;RP11-745A24.1;RP11-745A24.2;RP11-745A24.3;ICE2;RP11-82L7.4;RORA-AS1	0;0;0;0;0;262283;3944
ORF1_1_183089698_183091799_183167430_183172905_FF	0.026056839	1	1.398397306	LAMC1;RP11-181K3.4;RNU6-41P;LAMC2	0;0;107381;13334
ORF1_15_80632363_80638226_80872520_80879077_FF	0.011774113	1	1.403537255	RNU6-380P;ABHD17C;RP11-28H5.2;CEMIP;MIR549A;ARNT2;RP11-351M8.2	0;0;0;0;0;34427;17114
ORF1_1_201422030_201423605_201477609_201480715_FR	0.028280225	1	1.406485751	TNNI1;RP11-134G8.2;RP11-134G8.10;PHLDA3;LAD1;CSRP1	0;0;0;0;22423;2816
ORF1_3_149353577_149357857_149371594_149373294_FF	0.02260691	1	1.43594605	RP11-2G17.1;TM4SF1;TM4SF18;TM4SF1-AS1	0;0;19164;4485
ORF1_4_15897457_15905803_16069234_16073831_RR	0.038858138	1	1.437869828	FGFBP1;FGFBP2;PROM1;RP11-442P12.1;TAPT1	0;0;0;31518;86675
ORF1_19_50974727_50980421_50998297_50999377_RR	0.042151814	1	1.443524563	CTB-147C22.9;KLK7;KLK8;KLK9;KLK6;KLK9	0;0;0;0;5055;3735
ORF1_7_155235541_155238765_155312417_155316079_RR	0.003050624	1	1.447547802	AC099552.2;AC144652.1;INSIG1;RP11-1430O6.1;BLACE	0;0;0;11691;40907
ORF1_3_149353577_149357857_149401952_149407044_FF	0.017644893	1	1.451791344	RP11-2G17.1;TM4SF1;TM4SF1-AS1;RP11-278L15.2;TM4SF18;RP11-167H9.1	0;0;0;0;19164;26001
ORF1_3_149353577_149357857_149444838_149449455_FF	0.03660239	1	1.458921282	RP11-2G17.1;TM4SF1;TM4SF1-AS1;RP11-278L15.2;RP11-167H9.1;TM4SF18;RP11-278L15.3	0;0;0;0;0;19164;12728
ORF1_3_149353577_149357857_149619906_149622912_FR	0.024788664	1	1.465695938	RP11-2G17.1;TM4SF1;TM4SF1-AS1;RP11-278L15.2;RP11-167H9.1;RP11-278L15.3;RP11-278L15.4;TM4SF4;RP11-278L15.6;WWTR1;RNU6-1098P;TM4SF18;WWTR1-IT1	0;0;0;0;0;0;0;0;0;0;19164;26086
ORF1_15_80632363_80638226_80854680_80855742_FR	0.017368384	1	1.467431431	RNU6-380P;ABHD17C;RP11-28H5.2;CEMIP;MIR549A;ARNT2;RP11-351M8.2	0;0;0;0;0;34427;40449

ORF1_10_109690786_109701852_109746831_109753103_RR	0.00385767	1	1.471140831	RP11-566F5.1;RP11-451M19.2	9746;54549
ORF1_15_60380602_60382233_60406419_60409523_FR	0.044353798	1	1.48369954	ANXA2;RP11-745A24.1;RP11-745A24.2;RP11-745A24.3;RP11-82L7.4;ICE2	0;0;0;0;262283;10087
ORF1_10_109643643_109648810_109689464_109690786_RF	0.032705715	1	1.491346788	RP11-566F5.2;RP11-566F5.1;BTF3P15;RP11-451M19.2	0;0;108654;116866
ORF1_8_124140618_124142075_124265511_124269895_FF	0.000427397	1	1.49671285	FER1L6-AS2;RP11-959I15.1;ARF1P3;RP11-37N22.1;RNU6-756P;FER1L6;RP11-383J24.1	0;0;0;0;0;20558;1789
ORF1_8_124140618_124142075_124265511_124269895_FR	0.000400972	1	1.50060855	FER1L6-AS2;RP11-959I15.1;ARF1P3;RP11-37N22.1;RNU6-756P;FER1L6;RP11-383J24.1	0;0;0;0;0;20558;1789
ORF1_12_114994024_114996554_115050798_115060432_RR	0.047270365	1	1.503090999	RP11-25E2.1;RP11-411G2.1	16894;64090
ORF1_12_13036781_13040008_13175978_13179722_FR	0.041994439	1	1.522665349	RP11-377D9.3;KIAA1467;GSG1;RP11-377D9.1;EMP1	0;0;0;15176;16995
ORF1_8_124140618_124142075_124255305_124259479_FR	0.00118911	1	1.531476568	FER1L6-AS2;RP11-959I15.1;ARF1P3;RP11-37N22.1;FER1L6;RNU6-756P	0;0;0;0;20558;1168
ORF1_6_52352466_52355042_52491395_52492481_RF	0.000357245	1	1.53527918	PAQR8;EFHC1;MCM3;TRAM2	0;0;67586;4922
ORF1_1_201477609_201480715_201569360_201570965_RF	0.024749903	1	1.538252027	CSRP1;RP11-134G8.7;RP11-134G8.5;RP11-134G8.6;PHLDA3;NAV1	0;0;0;0;8373;51921
ORF1_10_119192489_119198194_119298530_119302992_FF	0.003201321	1	1.580687696	GRK5;GRK5-IT1;RN7SL749P;PRDX3;RP11-79M19.2	0;0;0;13657;27242
ORF1_6_52356722_52357908_52491395_52492481_RF	0.001279738	1	1.580804921	PAQR8;EFHC1;MCM3;TRAM2	0;0;71842;4922
ORF1_10_109689464_109690786_109832309_109837694_FF	0.008521447	1	1.59469172	RP11-451M19.2;RP11-566F5.1;XPNPEP1	0;8424;27073
ORF1_2_239167509_239171067_239271874_239274610_FR	0.03243505	1	1.609376076	HDAC4;AC017028.10;MIR4269	0;5153;30853
ORF1_1_201477609_201480715_201619157_201622754_RR	0.04315263	1	1.627918379	CSRP1;RP11-134G8.7;RP11-134G8.5;RP11-134G8.6;PHLDA3;NAV1	0;0;0;0;8373;132

ORF1_2_239167509_239171067_239257200_239261035_FF	0.025608121	1	1.646520395	HDAC4;AC017028.10;MIR4269	0;5153;44428
ORF1_2_172147393_172149085_172242412_172244329_RR	0.021766207	1	1.692796813	DLX2-AS1;AC104088.1	37412;70959
ORF1_10_109689464_109690786_109704546_109706912_FF	0.000906194	1	1.821797845	RP11-566F5.1;RP11-451M19.2	8424;100740
ORF1_12_13036781_13040008_13165147_13171938_FR	0.016339314	1	1.893256678	RP11-377D9.3;KIAA1467;GSG1;RP11-377D9.1;EMP1	0;0;0;15176;24779
ORF1_6_52347474_52349931_52491395_52492481_RF	0.000181646	1	1.966441186	PAQR8;EFHC1;MCM3;TRAM2	0;0;62594;4922
ORF1_1_201477609_201480715_201512879_201514970_RR	0.045274534	1	1.994299134	CSRP1;RP11-134G8.7;PHLDA3;RP11-134G8.5	0;0;8373;3734
ORF1_8_124155540_124159139_124255305_124259479_RR	0.021491592	1	2.551434124	FER1L6-AS2;RP11-37N22.1;ARF1P3;RNU6-756P	0;0;3108;1168
ORF1_15_100569072_100570377_100735444_100742675_FF	0.005708348	1	2.558915003	LINS;ASB7;Y_RNA;RP11-192M23.1;RP11-66B24.5;RNU6-181P;AC087762.1	0;0;0;0;0;8281;40796
