# Supplementary material for: A New Blood-Based Epigenetic Diagnostic Biomarker Test (EpiSwitch®® NST) with High Sensitivity and Positive Predictive Value for Colorectal Cancer and Precancerous Polyps
Source: Cancers (Basel). 2025 Feb 4;17(3):521. doi: 10.3390/cancers17030521 (PMC11816175; doi:10.3390/cancers17030521)
Supplement: Supplementary file 1 [file cancers-17-00521-s001.zip › Supplementary table S2 Four common markers.pdf]

Supplementary Table S2. Four common markers in *SGK223, ABCA4, THSD4*, and *SMAD3* genes shared between CRC and polyp classifiers.

Model markers	Array marker	Probe sequence	P.Value	adj.P.Val	FC	Gene	GeneDist
obd156_q1245_q1247	ORF1_8_8307248_8309141_8529093_8530943_RF	CAATAATTCATTCTTCTTCATCAGTCCTTCGAACTCCTGACTCAGGAGATCTATCCACCT	0.0161572	1	-1.342913514	SGK223;CTA-398F10.1;CTA-398F10.2;FAM86B3P;CTD-3023L14.3	0;0;0;62384;24796
obd156_q1217_q1219	ORF1_1_94060570_94064104_94081020_94084795_RF	TCTTGCCGGGAGTACTCTTCAAACTCCTTCGACATGATGGAGAAGCTGTCCAGGAACCAG	0.0000011	0.000163	1.535480941	ABCA4;RP5-837O21.2;RP11-78O9.1	0;125327;60317
obd156_q1297_q1299	ORF1_15_71449255_71457687_71567140_71571578_RR	GTACTGAATAATAGTGTATGTGTTTATGTCGACTGTACTGGCGGACCCTATAAGAGGCAG	6.85E-06	0.000421	1.462480028	THSD4;RP11-1123I8.1;RP11-592N21.2;AC104938.1	0;0;100785;201033
obd156_q1225_q1227	ORF1_15_67079527_67081854_67195948_67198335_RF	ATCTGTCCCAATCCTTTATCCTTCTAGCTCGAGTCAGCAGTGTTGACTGTTAGCAAATCA	1.8E-07	7.03E-05	1.652685053	SMAD3;RP11-342M21.2;RP11-798K3.2;AAGAB	0;0;20275;2699
