# Supplementary material for: A New Blood-Based Epigenetic Diagnostic Biomarker Test (EpiSwitch®® NST) with High Sensitivity and Positive Predictive Value for Colorectal Cancer and Precancerous Polyps
Source: Cancers (Basel). 2025 Feb 4;17(3):521. doi: 10.3390/cancers17030521 (PMC11816175; doi:10.3390/cancers17030521)
Supplement: Supplementary file 1 [file cancers-17-00521-s001.zip › Suppl table S3 Pathway analysis for the 8 Colorectal classifier markers.pdf]

Supplementary Table S3. Pathway analysis for the eight colorectal classifier markers; the table shows top pathways from the EpiSwitch 3D genome mapping app, and the data are ranked by score (-log2 of hypergeometric *p*-value)

Pathway	PathwaySource	PathwaySize	Input_list	Overlap	Score	V1	V2	V3	V4
Validated_targets_of_cMYC_transcriptional_activation	NCI	80	24	2	7.94	SMAD3	NDUFAF2		
TGF-beta signaling pathway	KEGG	95	24	2	7.459	SMAD3	THSD4		
Bile Acid Metabolism	Hallmark/Age	113	24	2	6.978	ABCA4	GCLM		
Colon (non-specific) lesion	DisGeNet	7	24	1	6.904	SMAD3			
Cell adhesion molecules	KEGG	149	24	2	6.219	CLDN23	CD58		
Arsenic.metabolism.and.reactive.oxygen.species.generation	Wiki	12	24	1	6.131	ATP1A1			
RHO GTPase Cycle	Reactome	442	24	3	6.115	MYO9A	ARHGAP29	PRAG1	
Epithelial.to.mesenchymal.transition.in.colorectal.cancer	Wiki	163	24	2	5.975	SMAD3	CLDN23		
Response To Metal Ions	Reactome	15	24	1	5.811	CSRP1			
GenAge_2023	Hallmark/Age	308	24	2	4.303	ERCC8	GCLM		
Nucleotide excision repair	KEGG	48	24	1	4.163	ERCC8			
Reactive Oxygen Species Pathway	Hallmark/Age	50	24	1	4.106	GCLM			
Multiple polyps	DisGeNet	62	24	1	3.806	SMAD3			
Adenomatous Polyposis Coli	DisGeNet	392	24	2	3.703	AAGAB	SMAD3		
Chromosomal.and.microsatellite.instability.in.colorectal.cancer	Wiki	74	24	1	3.561	SMAD3			
Adenocarcinoma of colon	DisGeNet	267	24	1	1.877	CD58			
Colorectal Neoplasms	DisGeNet	1073	24	2	1.529	ABCA4	GCLM		
Colorectal Carcinoma	DisGeNet	2931	24	4	1.445	ABCA4	CD58	GCLM	SMAD3
Malignant tumor of colon	DisGeNet	2001	24	3	1.42	AAGAB	GCLM	SMAD3	
Colon Carcinoma	DisGeNet	2091	24	3	1.326	AAGAB	CSRP1	SMAD3	
Colorectal Cancer	DisGeNet	3298	24	4	1.164	GCLM	ABCA4	CD58	SMAD3
Colonic Neoplasms	DisGeNet	778	24	1	0.739	SMAD3			
